# Systematic re-review of WASH trials to assess women’s engagement in intervention delivery and research activities

**DOI:** 10.1038/s44221-024-00299-2

**Published:** 2024-09-06

**Authors:** Bethany A. Caruso, April M. Ballard, Julia Sobolik, Madeleine Patrick, Janice Dsouza, Sheela S. Sinharoy, Oliver Cumming, Jennyfer Wolf, Isha Ray

**Affiliations:** 1 Hubert Department of Global Health, Rollins School of Public Health, Emory University, Atlanta, GA USA; 2 Gangarosa Department of Environmental Health, Rollins School of Public Health, Emory University, Atlanta, GA USA; 3 Department of Disease Control, Faculty of Infectious Tropical Disease, London School of Hygiene and Tropical Medicine, London, UK; 4 Department of Environment, Climate Change and Health, World Health Organization, Geneva, Switzerland; 5 Energy and Resources Group, University of California, Berkeley, CA USA

**Keywords:** Development studies, Environmental social sciences, Infectious diseases

## Abstract

Water, sanitation and hygiene (WASH) interventions significantly reduce health risks in low- and middle-income countries. Many rely on women, but the extent of women’s engagement remains undocumented. Here we conducted a re-review of papers from two systematic reviews that assessed the effectiveness of water, sanitation and/or handwashing with soap interventions on diarrhoeal disease and acute respiratory infections to assess women’s roles in WASH research and intervention activities. A total of 133 studies were included. Among studies that specified gender, women were the most sought-after group for engagement in research (*n* = 91/132; 68.9%) and intervention (*n* = 49/120; 40.8%) activities. Reporting time burden for research (*n* = 1; 1%) and intervention activities (*n* = 3; 2.5%) was rare. All interventions were classified as gender unequal (36.7%) or gender unaware (63.3%) according to the World Health Organization Gender Responsiveness Assessment Scale, indicating exploitative engagement. Women play a critical but instrumentalized role in WASH, and both research and interventions need to change to enable, and not hinder, gender equality.

## Abstract (web summary)

This systematic re-review of water, sanitation and hygiene trials found that women play a critical but instrumentalized role in research and intervention activities. Research and interventions need to change to enable, and not hinder, gender equality.

## Main

Water, sanitation and hygiene (WASH) services are foundational to human health and well-being. The results from two recent systematic reviews found that WASH services can significantly reduce the risk of diarrhoea and acute respiratory infections (ARIs) in low- and middle-income countries^1,2^. Recent disease burden estimates suggest that 1.4 million deaths and 74 million disability-adjusted life years were attributable to unsafe WASH in 2019^3^. However, due to a paucity of evidence, these estimates do not account for multiple other health outcomes related to WASH, including bodily injury, violence and stress^3^, which are often of particular concern for women and girls^4–7^.

In 2022, an estimated 27% of the global population (2.2 billion) lacked access to safely managed drinking water services, 43% (3.5 billion) to safely managed sanitation services and 25% (2 billion) to basic hygiene services^8^. As women and girls play a central, and sometimes outsized^9^, role in managing household WASH resources, these inadequate conditions place considerable burdens on them^4^. Due to gender norms, women and girls often bear responsibility for household WASH: time-consuming and physically arduous activities such as water fetching, latrine cleaning and keeping children clean^9–13^. While these activities may produce improvements in overall health, they demand women’s time and energy, limit opportunities^14^ and may result in risks to their own health and safety^4,15^. They could, therefore, perpetuate gender inequity^16^.

While these unpaid burdens and norms have been acknowledged, they also have been exploited by research and practice initiatives^15^, where women are often intentionally targeted by those delivering WASH programmes as key instruments for their success. For example, women have been trained to carry out water treatment, safe child faeces management and hand hygiene promotion, largely justified by the expected benefits for child health outcomes^12,17–19^. Yet assessment of the extent, and impact, of women’s engagement in WASH programmes for public health on women’s own health and well-being has been limited. A recent review of adoption of point-of-use chlorination for treating household drinking water found that most interventions deliberately targeted women to perform water treatment tasks, leveraging their household water management and caregiving roles. The time burden associated with this work was often reported to be a barrier to use but was seldom quantified^18^. Similarly, women are routinely expected to participate in research activities as part of large-scale WASH evaluations to provide detailed data about family members’ behaviours (for example, defecation practices)^19^ and health (for example, diarrhoea)^20–22^. These examples point to the need for comprehensive assessment of the central part that women play in health-related WASH research and practice.

Despite heavily involving women, rarely have WASH interventions and evaluations been designed and delivered specifically to improve or even understand their impact on women’s lives. WASH interventions should, however, be evaluated to understand whether and how potential burdens and benefits from these interventions have been distributed, and whether and how participants’ engagement reinforces existing gender roles. While some WASH interventions, such as household water treatment, place demands on women, others could relieve them. For example, piped water systems or passive chlorination devices may not only reduce child illness, they also could eliminate the time and labour required to fetch and treat water and the time, financial and psycho-social costs of caring for sick household members. Yet, these co-benefits are rarely assessed^23^.

The aim of this re-review is to assess how women are engaged in health-related WASH research and intervention activities. To do so, we conducted a re-review of papers from two recent systematic reviews published in *The Lancet* that assessed effectiveness of water, sanitation and/or handwashing with soap interventions on diarrhoeal disease^1^ and ARIs^2^. Specifically, in eligible studies we (1) identify the gender of the individual(s) engaged in research and intervention activities; (2) determine whether time required for engagement was reported and, if so, compensated; (3) discern whether additional intervention impacts specific to women were assessed; and (4) characterize intervention engagement overall and by intervention type using the World Health Organization (WHO) Gender Responsiveness Assessment Scale (GRAS)^24^.

## Assessing gender responsiveness in interventions

**Fig. 1:**
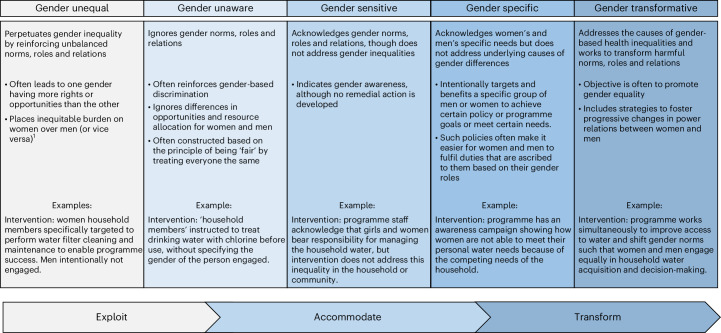
The GRAS and application to WASH interventions. The GRAS was developed by the WHO^24^ to assess health programmes, policies and interventions, depicting those approaches that should be avoided (gender unequal and gender blind) to those that are more desirable (gender sensitive, gender specific and gender transformative). In 2014, Pederson et al. modified the GRAS by adding the labels at the bottom to show that approaches aligned with the noted categories can exploit, accommodate or transform gender inequities, depending on how they are designed and delivered. The figure depicted here leverages the WHO GRAS and its modification by Pederson et al. (2014), changes one label from ‘gender blind’ to ‘gender unaware’ based on a recent critique (MacArthur^26^), builds upon the definition of gender unequal and provides hypothetical WASH examples along the scale. ^1^This portion of the ‘gender unequal’ definition was expanded upon by the co-authors.

The WHO GRAS^24^ for assessing gender responsiveness in health interventions, policies and programmes, and its elaboration by Pederson et al.^25^ is well suited for assessing intervention engagement. The WHO GRAS presents a spectrum of five gender approaches, from those that should be avoided (gender unequal and gender blind) to those that are more desirable (gender sensitive, gender specific and gender transformative). The modification by Pederson et al.^25^ includes all five approaches and also shows how health programmes, policies and interventions along these various levels can exploit, accommodate or transform gender inequities, depending on how they are designed and delivered. A recent iteration of the scale critiques the use of the term ‘gender blind’ and collapses the gender-unequal and gender-blind categories into one (‘gender insensitive’)^26^. We feel that these two categories are distinct and critical to retain for our analysis. Our slightly modified scale changes the term ‘gender blind’ to ‘gender unaware’, includes the definitions of all five categories as presented by the WHO^24^ and Pederson et al.^25^, builds upon the definition of gender unequal and provides hypothetical WASH examples along the scale (Fig. 1).

## Reporting sex-disaggregated outcome data was rare

We assessed all 150 studies included in the 2 reviews: 14 were duplicates and 3 were excluded due to language (Chinese, Danish and French), resulting in a final sample of 133 studies (Supplementary Fig. 1, flow diagram). We identified one intervention that appeared in two studies: one study^27^ was included in the diarrhoea systematic review and the other^28^ was in the ARI systematic review. As these papers could have engaged individuals differently in the research activities assessing their focal outcomes (diarrhoea and ARI) and because each could have described individual engagement in intervention activities differently, we elected to retain both and treat them independently. Studies with interventions that focused solely on drinking water (*n* = 64; 48.1%) or hygiene (*n* = 46; 34.6%) were the most common. Most studies took place in rural settings (*n* = 80; 60.2%) and in Asia (*n* = 51; 38.3%) and Africa (*n* = 43; 32.3%). Only eight (6.0%) studies presented sex-disaggregated outcome data (Table 1). Supplementary Table 1 presents a list of included studies and key characteristics.

**Table 1:** Summary information about included studies (*N* = 133)

	*n*	%
**Study source**		
Diarrhoea review only	107	80.5%
ARI review only	12	9.0%
Both diarrhoea and ARI	14	10.5%
**WASH focus of each study’s intervention**		
Water only	64	48.1%
Sanitation only	8	6.0%
Hygiene only	46	34.6%
Water, sanitation and hygiene	4	3.0%
Water and sanitation	4	3.0%
Water and hygiene	5	3.8%
Sanitation and hygiene	2	1.5%
**Total study intervention components that address water**^**a**^	77	57.9%
**Total study intervention components that address sanitation**^**a**^	18	13.5%
**Total study intervention components that address hygiene**^**a**^	57	42.9%
**Population for which the study intervention was seeking to improve the primary outcome**		
Children ≤5 years	111	83.5%
Other children^b^	10	7.5%
All ages	12	9.0%
**Study disaggregated primary outcome data by sex**	8	6.0%
**Study region**		
Africa	43	32.3%
Asia	51	38.3%
Europe	3	2.3%
Latin America and Caribbean	25	18.8%
Middle East	4	3.0%
North America	6	4.5%
Oceania	1	0.8%
**Study setting**		
Rural	80	60.2%
Urban	40	30.1%
Peri-urban	1	0.8%
Mixed	12	9.0%
**Study subsetting**		
Domestic	116	87.2%
Institutional	16	12.0%
Both domestic and institutional	1	0.8%

^a^May be more than one intervention component for water, sanitation and/or hygiene per study (for example, water treatment and storage).

^b^Includes children from fourth to fifth grade, children 5–15 years old, children less than 7 years old, children 9–11 years old, children ≤7 years old, children >5 years old, kindergarten children and school-aged children.

## Research activities largely depended on women

Individual-level participation in research activities was almost universal. Nearly all studies (*n* = 132; 99.2%) included at least one research activity that necessitated individual-level participation (Table 2). Of those, 92 (69.7%) studies engaged multiple groups in research activities and over half (*n* = 89; 67.4%) did not clearly specify who was engaged for at least one research activity. Overall, among participants specified, women were the most frequently engaged, followed by children. Women were reported to be engaged in 91 (68.9%) studies for research activities and were the only such group in 14 (10.6%) studies. The most common research activities that women were engaged in were surveys (for example, baseline and endline) (*n* = 63; 47.7%), diarrhoea recall (independent of other surveys) (53; 40.2%), behaviour/practice recall (independent of other surveys) (18; 13.6%) and observation (18; 13.6%) (Table 3). Children were engaged in 47 (35.6%) studies and were never the only participating group. Their participation was most sought for biological specimens, including stool or rectal swabs (15; 11.4%) and sera samples (9; 6.8%). Only two (1.5%) studies reported engaging men for any research activities (survey and qualitative research).

**Table 2:** Assessment of research and intervention engagement in included studies (*N* = 133)

	*n*	%
**Studies with research activities that involved a person for data collection**	132	99.2%
Only engaged women	14	10.6%
Only engaged men	0	0.0%
Only engaged women or men^a^	2	1.5%
Only engaged other specified individuals^b^	7	5.3%
Only engaged unspecified individuals	17	12.9%
Engaged multiple groups	92	69.7%
Total engaging women	91	68.9%
Total engaging men	2	1.5%
Total engaging women or men^a^	8	6.1%
Total engaging children	47	35.6%
Engaged girl and boy children (*n* = 47)	14	29.8%
Engaged unspecified children (*n* = 47)	37	70.2%
Total engaging other specified individuals^b^	24	18.2%
Total engaging unspecified individuals	89	67.4%
**Individual who reported study’s focal outcome (*n*** **=** **131)**^**c**^		
Women (mothers)	83	63.4%
Children	3	2.2%
School/daycare staff	7	5.3%
Parents	8	6.1%
Multiple types of people (doctors and so on)	2	1.5%
Unspecified	28	21.4%
**Studies that reported time required of participant for research activities**^**d**^	1	0.8%
**Studies that reported providing compensation for research activity engagement**	5	3.8%
**Studies that reported additional outcomes**	111	83.5%
Specific to women (*n* = 111)	16	14.4%
Specific to men (*n* = 111)	4	3.6%
Specific to children (*n* = 111)	60	54.1%
Specific to other populations (*n* = 111)^e^	92	82.9%
**Studies with intervention activities that necessitated involvement of a person**	120	90.2%
Only targeted women	21	17.5%
Only targeted men	0	0.0%
Only targeted women or men^a^	0	0.0%
Only targeted children	2	1.7%
Targeted girl and boy children (*n* = 2)	1	50.0%
Targeted unspecified children (*n* = 2)	1	50.0%
Only targeted other specified individuals^f^	10	8.3%
Only targeted unspecified individuals	39	32.5%
Targeted multiple groups	48	40.0%
Total targeting women	49	40.8%
Total targeting men	2	1.7%
Total targeting women or men	1	0.8%
Total targeting children	20	16.7%
Targeted girl and boy children (*n* = 20)	7	35.0%
Targeted unspecified children (*n* = 20)	13	65.0%
Total targeting other specified individuals^f^	31	25.8%
Total targeting unspecified individuals	76	63.3%
**Studies that reported time required of participant for intervention activities**^**d**^	3	2.5%
**Studies that reported providing compensation for intervention activity engagement**	13	10.8%
**Studies with intervention communications referencing shame, honour, upholding gender norms**	10	8.3%

^a^For interventions or research activities engaging or targeting ‘women or men’, these studies specifically indicated that either adult women or men were targeted.

^b^Examples of ‘other specified individuals’ include daycare administrators and staff, teachers and key informants.

^c^For two studies, diagnostic tests or medical records were used to retrieve data on the focal outcome, hence *n* = 131.

^d^Studies only counted if the information reported was clear and specific. Studies were considered to not have reported time if information provided was unclear, not specific enough or not reported at all.

^e^Examples of ‘other populations’ include daycare staff, household members, parents as a unit, households as a unit and schools as a unit.

^f^Examples of ‘other specified individuals’ include daycare administrators and staff, teachers and field-based staff.

**Table 3:** Summary of research activities by population engaged^a^

	Targeted women		Targeted men		Targeted women and/or men		Targeted children		Targeted other specified individuals^b^		Targeted unspecified individuals	
	*N*	%	*N*	%	*N*	%	*N*	%	*N*	%	*N*	%
Survey (for example, baseline, midline or endline)	63	47.7	1	0.8	7	5.3	5	3.8	18	13.6	60	45.5
Diarrhoea recall surveys (independent of other surveys)	53	40.2	0	0.0	2	1.5	2	1.5	4	3.0	24	18.2
Water behaviour/practices recall (independent of other surveys)	18	13.6	0	0.0	0	0.0	0	0.0	2	1.5	10	7.6
Observation (unstructured or structured)	18	13.6	0	0.0	0	0.0	5	3.8	7	5.3	28	21.2
Unannounced drop in visits	3	2.3	0	0.0	0	0.0	0	0.0	0	0.0	11	8.3
Qualitative research	11	8.3	1	0.8	0	0.0	0	0.0	3	2.3	0	0.0
Anthropometric measurements	5	3.8	0	0.0	0	0.0	2	1.5	0	0.0	2	1.5
Hand rinses	2	1.5	0	0.0	0	0.0	2	1.5	0	0.0	1	0.8
Stool or rectal swab collection	0	0.0	0	0.0	1	0.8	15	11.4	2	1.5	3	2.3
Sera sample	1	0.8	0	0.0	0	0.0	9	6.8	0	0.0	1	0.8
Nasal swabs	0	0.0	0	0.0	0	0.0	2	1.5	0	0.0	1	0.8
School absence logs	1	0.8	0	0.0	0	0.0	2	1.5	4	3.0	1	0.8
Case record forms about illnesses and/or school absences	11	8.3	0	0.0	0	0.0	2	1.5	9	6.8	5	3.8
Environmental swabs	1	0.8	0	0.0	0	0.0	0	0.0	0	0.0	3	2.3
Water testing (residual chlorine, total coliforms, *Escherichia coli* or faecal coliforms)	6	4.5	0	0.0	1	0.8	0	0.0	2	1.5	55	41.7

^a^Many studies involved multiple research activities, so the total number of activities per group may be greater than the total number of studies (*n* = 132).

^b^Examples of ‘other specified individuals’ include daycare administrators and staff, teachers and key informants.

The majority of studies depended on women to report the primary outcome (for example, child diarrhoea), yet few reported additional outcomes related to women’s own health or co-benefits (if any) such as time savings. In 83 (63.4%) studies, women—specifically mothers—were noted to have reported the study’s focal outcome. Most studies (111; 83.5%) reported additional outcomes other than diarrhoea and/or ARI. These additional outcomes related mainly to children (*n* = 60; 54.1%). Almost all (*n* = 59; 98.3%) reported on well-being outcomes (for example, growth, parasitic infection or school absence) and 10 (16.7%) reported on programme-related outcomes (for example, hand hygiene or defecation behaviour). Sixteen studies (14.4%) reported outcomes specific to women; three reported outcomes related to women’s well-being (for example, childcare hours saved, satisfaction with sanitation and water fetching time), while the rest focused on programme-related compliance (for example, hand hygiene, water treatment behaviours and so on). Four (3.6%) studies reported outcomes specific to men; two focused on men’s well-being (for example, time fetching water and satisfaction with sanitation) and two on programmatic outcomes (for example, defecation behaviours). Only one study (0.8%) comprehensively reported how much time was required for participants to engage in the research activities^29^ and five (3.8%) studies compensated those engaged in research for their time (Table 2).

## Women most targeted to carry out intervention activities

The majority of interventions required individual-level participation and most depended on women. One hundred twenty studies (90.2%) included at least one intervention activity that necessitated individual-level participation. Of those, 48 (40.0%) studies included intervention activities that engaged multiple groups, but over half of the studies (*n* = 76; 63.3%) did not specify who was engaged. Among those studies that specified, women were the most targeted for engagement in intervention activities, followed by children. Forty-nine (40.8%) studies specifically targeted women for participation, including 21 (17.5%) that targeted only women (Table 2). Among intervention activities, women were the most targeted group, including for all water- (46; 38.3%), sanitation- (15; 12.5%), hygiene- (35; 29.2%) and health promotion-related (29; 24.2%) activities, which included WASH-related health education, water treatment and child faeces management, among other activities (Table 4). Twenty (16.7%) intervention activities targeted children, including 2 (1.7%) that only targeted children. Children were most engaged in activities focused on hygiene practices and education. Only two (1.7%) studies reported targeting men for any intervention activities (hygiene-related health education and health promotion). Table 4 summarizes all intervention activities by populations engaged.

**Table 4:** Summary of intervention activities by population engaged (*N* = 120)^a^

	Targeted women		Targeted men		Targeted women and men		Targeted children		Targeted other specified individuals^b^		Targeted unspecified individuals	
	*n*	%	*n*	%	*n*	%	*n*	%	*n*	%	*n*	%
**All water-related activities**	46	38.3	0	0.0	1	0.8	4	3.3	15	12.5	76	63.3
Boiling	0	0.0	0	0.0	0	0.0	0	0.0	0	0.0	1	0.8
Filtering	2	1.7	0	0.0	0	0.0	0	0.0	1	0.8	18	15.0
Chlorination	7	5.8	0	0.0	0	0.0	0	0.0	5	4.2	18	15.0
Other chemical treatment	2	1.7	0	0.0	0	0.0	0	0.0	0	0.0	2	1.7
Solar water disinfection (SODIS)	9	7.5	0	0.0	0	0.0	1	0.8	1	0.8	1	0.8
Flocculation alone	2	1.7	0	0.0	0	0.0	0	0.0	0	0.0	0	0.0
Other water safety (for example, safe water storage)	3	2.5	0	0.0	0	0.0	0	0.0	4	3.3	14	11.7
Water treatment— improved water source (piped water, standpipe or borehole)	2	1.7	0	0.0	0	0.0	0	0.0	0	0.0	4	3.3
Water-related health education	19	15.8	0	0.0	1	0.8	3	2.5	4	3.3	18	15.0
**All sanitation-related activities**	15	12.5	0	0.0	1	0.8	4	3.3	5	4.2	33	27.5
Sanitation construction and improvements (without marketing and campaign)	2	1.7	0	0.0	0	0.0	1	0.8	1	0.8	8	6.7
Community-led total sanitation (CLTS)	0	0.0	0	0.0	0	0.0	0	0.0	0	0.0	4	3.3
Sanitation marketing	0	0.0	0	0.0	0	0.0	0	0.0	0	0.0	2	1.7
Child faeces disposal	3	2.5	0	0.0	0	0.0	0	0.0	1	0.8	3	2.5
Toilet facility cleaning	0	0.0	0	0.0	0	0.0	0	0.0	0	0.0	0	0.0
Sanitation—total sanitation campaign	0	0.0	0	0.0	0	0.0	0	0.0	0	0.0	5	4.2
Sanitation-related health education	10	8.3	0	0.0	1	0.8	3	2.5	3	2.5	11	9.2
**All hygiene-related activities**	35	29.2	1	0.8	1	0.8	31	25.8	28	23.3	53	44.2
Hygiene—receiving supplies, handwashing facilities or stations (for example, tippy taps)	3	2.5	0	0.0	0	0.0	2	1.7	2	1.7	7	5.8
Tippy tap construction	1	0.8	0	0.0	0	0.0	0	0.0	0	0.0	0	0.0
Handwashing	5	4.2	0	0.0	0	0.0	2	1.7	7	5.8	13	10.8
Child handwashing	2	1.7	0	0.0	0	0.0	10	8.3	4	3.3	2	1.7
Hygiene-related health education	24	20.0	1	0.8	1	0.8	17	14.2	15	12.5	31	25.8
**All health promotion**	29	24.2	1	0.8	0	0.0	3	2.5	16	13.3	26	21.7
General health promotion–recipient	16	13.3	0	0.0	0	0.0	2	1.7	3	2.5	17	14.2
General health promotion–individual engaged as promoter	13	10.8	1	0.8	0	0.0	1	0.8	13	10.8	9	7.5

^a^Many studies involved multiple interventions activities, so the total number of activities per group may be greater than the total number of studies (*n* = 120).

^b^Examples of ‘other specified individuals’ include daycare administrators and staff, teachers and key informants.

Despite the near ubiquitous need for individual-level participation in the WASH interventions assessed, studies rarely reported time burden or compensation: only 3 (2.5%) studies reported the time burden of engaging in intervention activities and 13 (10.8%) reported providing compensation to individuals for their time (Table 2). From the information reported, ten (8.4%) studies explicitly referenced gender norms as an intentional part of their interventions; these referenced shame, honour or upholding traditional values (for example, campaigns that promoted handwashing as practiced by ‘good mothers’).

## All interventions were gender unequal or gender unaware

We classified all study interventions as either gender unequal (36.7%) or gender unaware (63.7%) (Table 5), categorizations that are termed exploitative by WHO and that are, by extension, not recommended.

**Table 5:** Gender responsiveness assessment of interventions in included studies by type and population engagement (*N* = 133)

	Did not require individual-level participation		Required individual-level participation		GRAS classification among those that required individual-level participation^a^			
					Gender unequal		Gender unaware	
	*n*	%	*n*	%	*n*	%	*n*	%
**Interventions (133)**	13	9.8%	120	90.2%	44	36.7%	76	63.3%
Water (*n* = 64)	8	12.5%	56	87.5%	19	33.9%	37	66.1%
Sanitation (*n* = 8)	4	50.0%	4	50.0%	0	0.0%	4	100.0%
Hygiene (*n* = 46)	0	0.0%	46	100.0%	17	37.0%	29	63.0%
Water, sanitation and hygiene (*n* = 4)	0	0.0%	4	100.0%	3	75.0%	1	25.0%
Water and sanitation (*n* = 4)	1	25.0%	3	75.0%	1	33.3%	2	66.7%
Water and hygiene (*n* = 5)	0	0.0%	5	100.0%	3	60.0%	2	40.0%
Sanitation and hygiene (*n* = 2)	0	0.0%	2	100.0%	1	50.0%	1	50.0%
**Type of water intervention** **(*n*** **=** **77)**	9	11.7%	68	88.3%	24	35.3%	44	64.7%
Improved, on premise, continuous supply (*n* = 1)	1	100.0%	0	0.0%	0	0.0%	0	0.0%
Improved, on premise, higher water quality (*n* = 2)	0	0.0%	2	100.0%	0	0.0%	2	100.0%
Improved, on premise (*n* = 9)	6	66.7%	3	33.3%	0	0.0%	3	100.0%
Improved, not on premise (*n* = 8)	2	25.0%	6	75.0%	2	38.6%	4	61.4%
Point-of-use treatment of water from unimproved water source or improved source not on premise (*n* = 57)	0	0.0%	57	100.0%	22	28.9%	35	46.1%
**Type of sanitation intervention** **(*n*** **=** **18)**	5	27.8%	13	72.2%	4	30.8%	9	69.2%
Sewer connection (*n* = 4)	4	100.0%	0	0.0%	0	0.0%	0	0.0%
Basic sanitation/improved sanitation (*n* = 14)	1	7.1%	13	92.9%	3	23.1%	10	76.9%
**Type of hygiene intervention** **(*n*** **=** **57)**	0	0.0%	57	100.0%	23	40.4%	34	59.6%
Promotion of handwashing with soap provision (*n* = 34)	0	0.0%	34	100.0%	10	29.4%	24	70.6%
Promotion of handwashing with no provision of soap (*n* = 23)	0	0.0%	23	100.0%	13	56.5%	10	43.5%
**Classification by group targeted for intervention engagement** **(*n*** **=** **120)**								
Specific to women (*n* = 21)					21	100.0%	0	0.0%
Specific to men (*n* = 0)					0	0.0%	0	0.0%
Specific to children (*n* = 2)					0	0.0%	2	100.0%
Specific to other specified individuals (*n* = 10)^b^					0	0.0%	10	100.0%
Specific to unspecified populations (*n* = 39)					0	0.0%	39	100.0%
Multiple populations (*n* = 48)					23	47.9%	25	52.1%

^a^The only categories listed are ‘gender unequal’ and ‘gender unaware’ because none of the other categories were represented.

^b^Examples of ‘other specified individuals’ targeted for intervention activities include daycare administrators and staff, teachers and field-based staff.

**Fig. 2:**
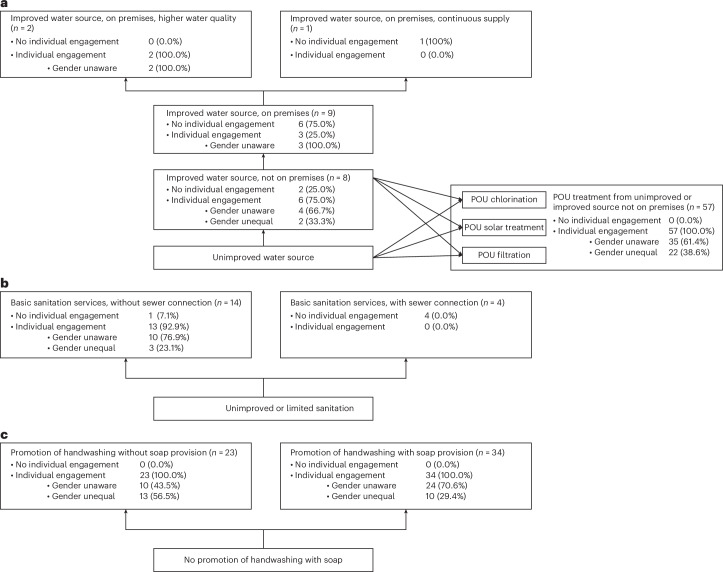
Exposure scenarios with GRAS classifications. **a**–**c**, Depicted exposure scenarios adapted from Wolf et al.^1^ who used the exposure scenarios to determine diarrhoea risk at the various WASH service levels. The authors concluded that higher service levels provided increased protection from diarrhoeal pathogens. We used the same exposure scenarios to determine whether and how gender responsiveness varied at the different service levels for water (**a**), sanitation (**b**) and hygiene (**c**). We found high levels of exploitative engagement at lower service levels. POU, point of use.

Of the 77 studies that included water interventions, 9 (11.7%) did not require any individual participation and among the remaining 68 that did, 24 (35.3%) are gender unequal and 44 (64.7%) are gender unaware (Table 5). Supplementary Table 2 presents examples of gender-unequal and gender-unaware water intervention activities. Disaggregating by different levels of drinking water services, 57 (74.0%) involved point-of-use water treatment for sources off premises, a low level of service according to the exposure scenario. Among these, 22 (38.6%) are categorized as gender unequal and 35 (61.4%) as gender unaware. The one intervention that provided improved, on premises, continuous water supply—one of the higher levels of water service represented by the exposure scenario—was not evaluated using the GRAS framework as it did not require individual-level involvement (Fig. 2).

Of the 18 studies that included sanitation interventions, five (27.8%) did not require any individual involvement and among the remaining 13 that did, all provided basic sanitation services without sewage connection with three (23.1%) categorized as gender unequal and 10 (76.9%) as gender unaware. (Table 5). The four interventions that provided basic sanitation with sewer connections were not evaluated using the GRAS framework as they did not require individual-level involvement (Fig. 2).

Of the 57 studies that included hygiene interventions, all required individual-level involvement and involved some type of hand hygiene promotion, though only 34 (59.6%) provided soap. Fewer interventions that provided soap were gender unequal (*n* = 10; 29.4%) than were gender unaware (*n* = 24; 70.6%). Conversely, a greater proportion of the hand hygiene promotion interventions that did not provide soap were gender unequal (*n* = 13; 56.5%) rather than gender unaware (*n* = 10; 43.5%) (Fig. 2 and Table 5).

For the two studies that reported different outcomes related to the same intervention^27,28^, the study reporting on diarrhoea as the focal outcome^27^ did not specify who was targeted for engagement in intervention activities, leading to a GRAS assessment of ‘gender unaware’. Conversely, the study reporting on ARI as the focal outcome^28^ did specify who was targeted for engagement in intervention activities, leading to a GRAS assessment of ‘gender unequal’.

## Discussion

In this re-review of 133 studies from two systematic reviews assessing effectiveness of water, sanitation and/or handwashing with soap interventions on diarrhoeal disease^1^ and ARIs^2^, we evaluated the reported engagement of individuals in the evaluation and delivery of WASH interventions. We find that, in many instances, the interventions that were the subject of these studies relied entirely on women as the agents of delivery and as the source of study data. WASH interventions were overwhelmingly gender unequal or gender unaware and therefore classified as gender exploitative under the GRAS framework (Fig. 1). Any costs to women’s own time or benefits to their own lives were rarely mentioned. Women appear to play a critical but purely instrumentalized role in advancing WASH. The often-unacknowledged role of women in the evaluation and implementation of health-related WASH studies has several unintended, yet detrimental, consequences that require change for WASH if it is to enable gender equality and not hinder it.

Perhaps the most insidious consequence of taking for free and for granted women’s time and cooperation in WASH is that it cements existing and unequal gender norms. Maintaining family health, which includes WASH tasks, is considered to be women’s work, and women’s labour is ‘understood’ to be of low value. When WASH implementers and researchers not only avoid playing an equalizing role, but actively exploit gender-unequal roles, then existing inequalities are reproduced^30^ or even strengthened^16^. These observations—that women perform unpaid WASH labour and that this renders the research itself exploitative—are not new; this bias has been described since the early 1980s^31–33^. Nonetheless, the exploitation of gender stereotypes and acceptance of numerous hours of unpaid labour by women has persisted. While occurring in both research and intervention delivery, the impact of engagement is probably quite different; the demand for women’s unpaid labour in intervention delivery, in particular, could be sustained indefinitely or even scaled up if deemed effective at improving child health. Moving forward, WASH programmes and interventions should be evaluated using the GRAS tool, or similar adaptations^26^, before implementation so those classified as exploitative can be re-designed or abandoned. Exploitative interventions should not be funded for evaluation.

Regardless of the effectiveness of the WASH interventions assessed, the full implementation costs have not been transparently acknowledged in evaluations or reflected in subsequent recommendations. Many WASH approaches, especially household-based approaches, are touted as ‘low cost’ by depending on women’s ‘free’ time and labour. These falsely low costs are routinely highlighted as a benefit for—and even made a stipulation by—policymakers and donors, who often demand evidence of cost-effectiveness, put caps on the total costs allowed for an intervention and restrict the types of allowable expenses (for example, participant compensation). We acknowledge that the extent and nature of compensation must be context specific so as not to place undue burdens on low-income communities and non-governmental organizations, and we recommend that WASH actors (1) budget appropriate compensation for those who shoulder the burdens of making these interventions ‘work’, (2) transparently report who is engaged and (3) rigorously evaluate participant time and opportunity costs. When reporting who is engaged, the Sex and Gender Equity in Research (SAGER) guidelines, which have been adopted by the WHO^34^, are a useful reference. Designed to eliminate gender data gaps, the SAGER guidelines provide recommendations for reporting disaggregated sex and gender data, and also encourage including sex and gender dimensions when designing research, collecting data and undergoing analyses^35^. Further, gender has been acknowledged as just one of many characteristics that intersect to contribute to inequalities related to WASH^36,37^, and therefore an intersectional lens is also relevant to identifying additional markers of identity that may compound experiences of inequality beyond gender alone^38^.

The gender-unequal or gender-unaware interventions were largely among interventions that represent lower levels of service, illuminating how these allegedly low-cost interventions not only demand ‘free’ labour, but extract this labour to provide services or promote approaches that are often inferior. Interventions at the lowest service levels often emphasize behaviour change and, as we and others^18^ have shown, most target women’s behaviour change. Yet behaviour change approaches are ‘generally the least effective type of intervention’^39^. Furthermore, ‘the need to urge behavioural change is symptomatic of failure to establish contexts in which healthy choices are default actions’^39^. As a result, the women conscripted to perform (or enforce) WASH behaviours are probably living in the least enabling environments and therefore may have little chance for impact despite their efforts. Failed behaviour-change interventions tend to be ascribed to poor ‘compliance’, which blames individuals—largely women—for intervention failure as opposed to the possible inappropriateness of the approach itself^40^. Our data show that factors that shape individual ability to adopt interventions (for example, time and finances)—which are useful to assess intervention appropriateness—were rarely documented. In contrast, the most common reported outcomes related to women were about their ‘compliance’ behaviours. Higher WASH service levels are critical for health^3^ and for establishing contexts that enable healthy choices including relieving women’s labour, saving energy costs and time and lowering stress.

WASH provision at higher service levels does tend to require less household work, but cannot guarantee that women will not be burdened or that their needs will be met. WASH approaches therefore need to be intentionally gender sensitive, at a minimum. The Joint Monitoring Programme for Water Supply, Sanitation and Hygiene service ladders, which function as the benchmark by which to evaluate the quality of WASH services, are notably gender unaware^41^ and therefore insufficient as the only benchmark. As an example, toilets can be categorized to be at the highest service level (safely managed) even if they lack a superstructure or a door because the ladder does not assess privacy. The global WASH community is already calling for a paradigm shift in how WASH services are delivered and evaluated^42^. Consistent with this call, we recommend that potential gender-related needs, burdens and benefits are formally included when assessing the quality of WASH services, as well as in WASH evaluations when assessing their effectiveness in preventing disease.

A shift is also needed in how evaluations of WASH interventions are conceived, conducted and communicated to prevent further gender exploitation. As with intervention delivery, studies are not always explicit about who is engaged in research activities, women are routinely engaged, compensation is rare and few report the time participation required. Women, in effect, act as unpaid research assistants. While there remain debates about research compensation^43^, researchers and donors should be deliberate about time required from research participants and justify compensation decisions transparently.

These conclusions are limited by the information reported in the papers assessed, did not consider studies that may have been published elsewhere, excluded evaluations in languages other than English or Spanish and may have a restricted sample because of the sources from which included studies were identified. Despite any potential sample limitations, our findings demonstrate a clear trend in how women have been engaged in research and intervention delivery, calling into question how women may have been engaged in other WASH intervention studies, whether seeking to improve health outcomes or not. The approach used herein can and should be used to interrogate other WASH research and interventions, regardless of whether they seek to improve health outcomes, and to any other research and intervention activities that rely on individuals to perform activities.

Our re-review nonetheless takes a gender lens to prominent studies used to determine intervention effectiveness on key health outcomes. This lens should be considered when assessing the health impacts of WASH interventions. Specifically, women have been critical to evaluation research and intervention delivery and yet are often invisible and undervalued in the public health literature. Greater awareness and reflexivity are needed within WASH research and practice to elevate and value gender equity alongside health impacts.

## Methods

We re-reviewed papers from two recent systematic reviews published in *The Lancet* that assessed effectiveness of water, sanitation and/or handwashing with soap interventions on diarrhoeal disease^1^ and ARIs^2^. The search terms, inclusion and exclusion criteria, risk of bias and GRADE (Grading of Recommendations, Assessment, Development and Evaluation) scores can be found in these two reviews and their supplementary appendices. The protocol is registered with PROSPERO (CRD42022346360). We report findings following the Preferred Reporting Items for Systematic Reviews and Meta-Analyses criteria (Supplementary Tables 3 and 4).

### Inclusion criteria and eligibility

All papers included in the two previously published systematic reviews were eligible for inclusion if published in English or Spanish. Studies were excluded if not in English or Spanish^1,2^.

### Data extraction

Two reviewers independently extracted data from each article using a common data extraction template in excel (extraction sheet available with the public dataset on Figshare at 10.6084/m9.figshare.25786638). To test the data extraction sheet and to ensure consistency in the process, reviewers first extracted data from the same five articles and compared their data. Any disagreements were discussed to ensure common understanding before official extraction was initiated. Once data had been extracted twice from each article, the two completed data extraction sheets were compared to identify any inconsistencies. If there was an inconsistency, one team member returned to the study to re-extract the relevant data.

To identify the gender of individuals engaged in research and intervention activities, reviewers first extracted data on whether intervention and evaluation activities required individual-level participation from the target households/communities. Among those that required individual-level participation, reviewers identified who was engaged (women, men, men and/or women, girls, boys, girls and/or boys, other specified and/or unspecified individuals/populations). The dataset provides more detail on terms used and categorization assumptions (for example, mother, caregiver or categorized as ‘women’). They further extracted data on the time required for engagement and compensation provided (if these were reported); who reported the study’s focal outcome (for example, child diarrhoea); if any additional intervention impacts specific to women, men, girls or boys were assessed; and if the intervention activities included messages that involved shame or honour (for example, establishing norms of ‘good’ parenting).

We used the adapted GRAS figure (Fig. 1) as a tool to assess gender responsiveness in those interventions that required individual-level participation. As with the extraction for the other variables, two reviewers independently reviewed intervention descriptions from the included studies and categorized them using the definitions noted in the figure. As categorization is more subjective than extraction for the other variables, any inconsistencies in categorization were reconciled through discussion with a third team member. Interventions with multiple components can have different GRAS categories for each component^24^, thus we categorized each water, sanitation and/or hygiene component in an intervention separately and provided an overall categorization of the intervention. We did not assess any non-WASH (for example, nutrition) intervention components.

### Analysis

We used R Studio v4.0.5 to generate descriptive statistics about which individuals were engaged in the research and interventions assessed; how they were engaged; what additional outcomes, if any, were evaluated; and how the interventions were categorized using the GRAS categories. We further organized the GRAS data by the WASH exposure scenarios presented by Wolf et al.^1^. These exposure scenarios were informed by the definitions and exposure levels of the service ladders created by the WHO/UNICEF Joint Monitoring Programme for Water Supply, Sanitation and Hygiene to assess progress against SDG targets 6.1 and 6.2, and were adapted based on available evidence. Wolf et al.^1^ used the exposure scenarios to determine diarrhoea risk at the various WASH service levels and concluded that higher service levels provided increased protection from diarrhoeal pathogens. We used the same exposure scenarios to determine whether and how gender responsiveness varied at the different service levels.

### Positioning and role of the funding source

The authors describe how their experiences and perspectives informed the re-review in Supplementary Text 1.

The funders of the study had no role in study design, data collection, analysis, interpretation, writing of the report or decision to publish.

## Supplementary information

Supplementary Information. Supplementary Fig. 1, Tables 1–4, Text 1 and References.

## Data Availability

All data are publicly available on Figshare at 10.6084/m9.figshare.25786638 (ref. ^44^).
