# Supplementary material for: Systematic re-review of WASH trials to assess women’s engagement in intervention delivery and research activities
Source: Nat Water. 2024 Sep 6;2(9):827–36. doi: 10.1038/s44221-024-00299-2 (PMC11412895; doi:10.1038/s44221-024-00299-2)
Supplement: Supplementary file 1 — Supplementary Fig. 1, Tables 1–4, Text 1 and References. [file 44221_2024_299_MOESM1_ESM.pdf]

nature water

Supplementary information https://doi.org/10.1038/s44221-024-00299-2

Systematic re-review of WASH trials to assess women's engagement in intervention delivery and research activities

In the format provided by the authors and unedited

Table of Contents

Supplementary Figure 1. Study Selection ______ 2
Supplementary Table 1: Included Studies and Key Characteristics ______ 3
Supplementary Table 2: Examples of gender-unequal and gender-unaware water, sanitation, and hygiene intervention activities from included studies ______ 13
Supplementary Table 3: Prisma Checklist for Abstracts ______ 14
Supplementary Table 4: Prisma Checklist for Reviews ______ 15
Supplementary Text 1: Reflexivity Statement ______ 18
Supplementary References 1. Full Citations for All Included Studies ______ 19

Supplementary Figure 1. Study Selection

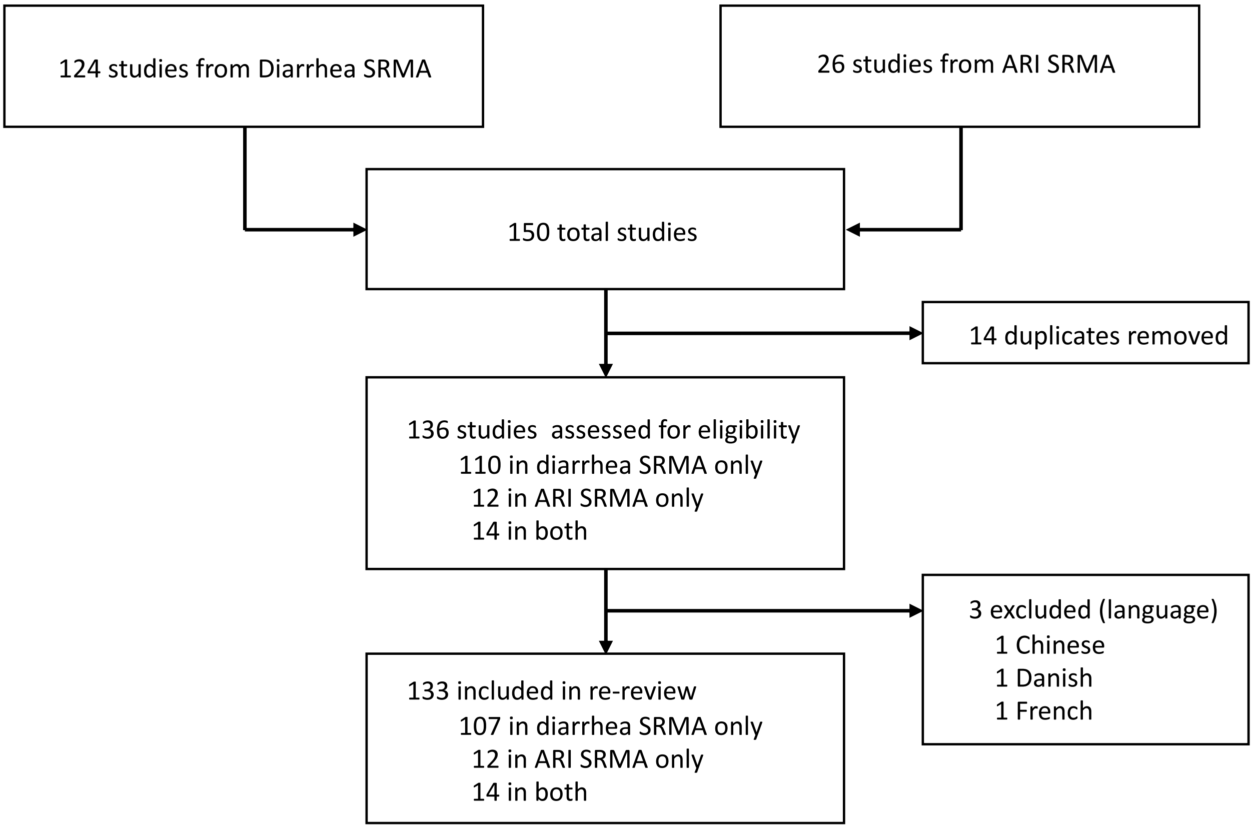

Supplementary Table 1: Included Studies and Key Characteristics

Study ID	SRMA source[a]	Int. Focus[b]	Int. Type[c]	Study region	Sex-disaggregated outcome data reported	Individuals targeted for intervention activities[d]	Individuals engaged for research activities[d]	GRAS overall	GRAS water int.	GRAS san. int.	GRAS hygiene int.
Ahmed 1993[1]	D	H	H.0	Asia	No	W	W, W or M, C	Gender unequal	NA	NA	Gender unequal
Aiken 2011[2]	D	W	W.5	Latin America	Yes	UI	UI	Gender unaware	Gender unaware	NA	NA
Alam 1989[3]	D	W	W.4	Asia	No	W	W, UI	Gender unequal	Gender unequal	NA	NA
Arnold 2009[4]	D, ARI	W, H	W.5, H.0	Latin America	No	W	W, C, UI	Gender unequal	Gender unequal	NA	Gender unequal
Arnold 2010[5]	D	W, S, H	W.3, S.2, H.0	Asia	No	W, C, UI	W, C, UI	Gender unequal	Gender unaware	Gender unaware	Gender unequal
Ashraf 2020[6]	ARI	H	H.1	Asia	Yes	W, UI	W, UI	Gender unaware	NA	NA	Gender unaware
Austin 1993[7]	D	W	W.5	Africa	No	W	W, C, UI	Gender unequal	Gender unequal	NA	NA
Aziz 1990[8]	D	W, S	W.4, S.2	Asia	No	W	W	Gender unequal	Gender unequal	Gender unequal	NA
Azor-Martinez 2020[9]	D	H	H.1	Europe	No	C, OSI	OSI	Gender unaware	NA	NA	Gender unaware
Ban 2015[10]	D, ARI	H	H.1	Asia	No	C, OSI	OSI	Gender unaware	NA	NA	Gender unaware
Bartlett 1988[11]	D	H	H.0	North America	No	OSI	C, OSI, UI	Gender unaware	NA	NA	Gender unaware
Begum 2020[12]	D	H	H.0	Asia	No	W	W	Gender unequal	NA	NA	Gender unequal
Bitew 2018[13]	D	W	W.5	Africa	No	W	W, UI	Gender unequal	Gender unequal	NA	NA

(Supplementary Table 1 Continued)

Study ID	SRMA source[a]	Int. Focus[b]	Int. Type[c]	Study region	Sex-disaggregated outcome data reported	Individuals targeted for intervention activities[d]	Individuals engaged for research activities[d]	GRAS overall	GRAS water int.	GRAS san. int.	GRAS hygiene int.
Black 1981[14]	D	H	H.0	North America	No	OSI	C, OSI	Gender unaware	NA	NA	Gender unaware
Boisson 2013[15]	D	W	W.5	Asia	No	UI	W, C, UI	Gender unaware	Gender unaware	NA	NA
Bowen 2007[16]	ARI	H	H.1	Asia	No	C, OSI	OSI	Gender unaware	NA	NA	Gender unaware
Briceño 2015[17]	D	S, H	S.2, H.1	Africa	No	W, UI	W, C, UI	Gender unequal	NA	Gender unaware	Gender unequal
Brown 2007[18]	D	W	W.5	Asia	Yes	UI	W, W or M, UI	Gender unaware	Gender unaware	NA	NA
Brown 2008[19]	D	W	W.5	Asia	Yes	UI	W, UI	Gender unaware	Gender unaware	NA	NA
Brown 2013[20]	D	W	W.3	Asia	No	None	W	NA	NA	NA	NA
Butz 1990[21]	D	H	H.0	North America	No	OSI	OSI	Gender unaware	NA	NA	Gender unaware
Carabin 1999[22]	D	H	H.0	North America	No	OSI	C, OSI, UI	Gender unaware	NA	NA	Gender unaware
Cha 2015[23]	D	W	W.4	Africa	No	W or M, UI	W, UI	Gender unaware	Gender unaware	NA	NA
Chard 2019[24]	ARI	H	H.1	Asia	No	C, UI	C, OSI, UI	Gender unaware	NA	NA	Gender unaware
Chase 2012[25]	D, ARI	H	H.0	Asia	No	W, OSI	W, C, OSI, UI	Gender unequal	NA	NA	Gender unequal
Chiller 2006[26]	D	W	W.5	Latin America	No	W	W, UI	Gender unequal	Gender unequal	NA	NA
Chongsuviv-atwong 1994[27]	D	W	W.3	Asia	No	None	W	NA	NA	NA	NA
Clasen 2004[28]	D	W	W.5	Latin America	No	OSI, UI	UI	Gender unaware	Gender unaware	NA	NA

(Supplementary Table 1 Continued)

Study ID	SRMA source[a]	Int. Focus[b]	Int. Type[c]	Study region	Sex-disaggregated outcome data reported	Individuals targeted for intervention activities[d]	Individuals engaged for research activities[d]	GRAS overall	GRAS water int.	GRAS san. int.	GRAS hygiene int.
Clasen 2005[29]	D	W	W.5	Latin America	No	UI	UI	Gender unaware	Gender unaware	NA	NA
Clasen 2006[30]	D	W	W.5	Latin America	No	UI	W, UI	Gender unaware	Gender unaware	NA	NA
Clasen 2014[31]	D	S	S.2	Asia	No	UI	W, C, UI	Gender unaware	NA	Gender unaware	NA
Conroy 1996[32]	D	W	W.5	Africa	No	C	W, OSI	Gender unaware	Gender unaware	NA	NA
Conroy 1999[33]	D	W	W.5	Africa	No	W	UI	Gender unequal	Gender unequal	NA	NA
Crump 2005[34]	D	W	W.5	Africa	No	UI	W, UI	Gender unaware	Gender unaware	NA	NA
Devoto 2011[35]	D	W	W.3	Africa	No	UI	W, UI	Gender unaware	Gender unaware	NA	NA
Dickinson 2015[36]	D	S	S.2	Asia	Yes	UI	W, M, C, UI	Gender unaware	NA	Gender unaware	NA
du Preez 2008[37]	D	W	W.5	Africa	No	UI	W, UI	Gender unaware	Gender unaware	NA	NA
du Preez 2010[38]	D	W	W.5	Africa	No	W	W, UI	Gender unequal	Gender unequal	NA	NA
du Preez 2011[39]	D	W	W.5	Africa	No	W	W, C, UI	Gender unequal	Gender unequal	NA	NA
Ercumen 2015[40]	D	W	W.1	Asia	No	None	W, C, UI	NA	NA	NA	NA
Ercumen 2015[41]	D	W	W.5	Asia	No	UI	W, UI	Gender unaware	Gender unaware	NA	NA
Fabiszewski de Aceituno 2012[42]	D	W	W.5	Latin America	No	UI	W, UI	Gender unaware	Gender unaware	NA	NA
Fagerli 2020[43]	D	W	W.5	Africa	No	UI	W, UI	Gender unaware	Gender unaware	NA	NA

(Supplementary Table 1 Continued)

Study ID	SRMA source[a]	Int. Focus[b]	Int. Type[c]	Study region	Sex-disaggregated outcome data reported	Individuals targeted for intervention activities[d]	Individuals engaged for research activities[d]	GRAS overall	GRAS water int.	GRAS san. int.	GRAS hygiene int.
Galiani 2009[44]	D	W	W.3	Latin America	No	None	UI	NA	NA	NA	NA
Galiani 2015[45]	D, ARI	H	H.0	Latin America	No	W, C, OSI, UI	W, C, UI	Gender unequal	NA	NA	Gender unequal
Garrett 2008[46]	D	W, S	W.5, S.2	Africa	No	OSI, UI	W, W or M	Gender unaware	Gender unaware	Gender unaware	NA
Gasana 2002[47]	D	W	W.4	Africa	No	UI	C, UI	Gender unaware	Gender unaware	NA	NA
Graf 2010[48]	D	W	W.5	Africa	No	OSI, UI	W, UI	Gender unaware	Gender unaware	NA	NA
Gruber 2013[49]	D	W	W.5	Latin America	No	OSI, UI	W, UI	Gender unaware	Gender unaware	NA	NA
Haggerty 1994[50]	D	H	H.0	Africa	No	W, UI	UI	Gender unequal	NA	NA	Gender unequal
Hammer 2016[51]	D	S	S.2	Asia	No	UI	W, C	Gender unaware	NA	Gender unaware	NA
Han 1989[52]	D	H	H.1	Asia	No	W, C	W	Gender unequal	NA	NA	Gender unequal
Harshfield 2012[53]	D	W	W.5	Latin America	Yes	UI	W or M	Gender unaware	Gender unaware	NA	NA
Hartinger 2016[54]	D, ARI	W, H	W.5, H.0	Latin America	No	W, UI	W, C, UI	Gender unequal	Gender unequal	NA	Gender unequal
Hashi 2017[55]	D	H	H.1	Africa	No	W	W, UI	Gender unequal	NA	NA	Gender unequal
Hill 2020[56]	D	W	W.5	Africa	No	W	W, C, UI	Gender unequal	Gender unequal	NA	NA
Huda 2012[57]	D, ARI	H	H.0	Asia	No	UI	UI	Gender unaware	NA	NA	Gender unaware
Humphrey 2019[58]	D, ARI	W, S, H	W.5, S.2, H.0	Africa	No	W, UI	W, C	Gender unequal	Gender unequal	Gender unequal	Gender unequal

(Supplementary Table 1 Continued)

Study ID	SRMA source[a]	Int. Focus[b]	Int. Type[c]	Study region	Sex-disaggregated outcome data reported	Individuals targeted for intervention activities[d]	Individuals engaged for research activities[d]	GRAS overall	GRAS water int.	GRAS san. int.	GRAS hygiene int.
Hunter 2010[59]	D	W	W.2	Latin America	No	OSI	UI	Gender unaware	Gender unaware	NA	NA
Hussam 2022[60]	ARI	H	H.1	Asia	No	UI	W, C, UI	Gender unaware	NA	NA	Gender unaware
Iijima 2001[61]	D	W	W.5	Africa	No	W, UI	UI	Gender unaware	Gender unaware	NA	NA
Jain 2010[62]	D	W	W.5	Africa	No	UI	W, UI	Gender unaware	Gender unaware	NA	NA
Jensen 2003[63]	D	W	W.4	Asia	No	OSI	W, UI	Gender unaware	Gender unaware	NA	NA
Kirby 2017[64]	D	W	W.5	Africa	No	UI	W, OSI	Gender unaware	Gender unaware	NA	NA
Kirby 2019[65]	D	W	W.5	Africa	No	UI	W, OSI	Gender unaware	Gender unaware	NA	NA
Kirchhoff 1985[66]	D	W	W.5	Latin America	No	W	W, UI	Gender unequal	Gender unequal	NA	NA
Klasen 2012[67]	D	W, S	W.3, S.1	Middle East	No	None	UI	NA	NA	NA	NA
Kolahi 2009[68]	D	S	S.1	Middle East	No	None	W	NA	NA	NA	NA
Kotch 1994[69]	D	H	H.1	North America	No	OSI	OSI	Gender unaware	NA	NA	Gender unaware
Kotch 2007[70]	D	H	H.1	North America	No	OSI	OSI	Gender unaware	NA	NA	Gender unaware
Kremer 2009[71]	D	W	W.4	Africa	No	UI	W, UI	Gender unaware	Gender unaware	NA	NA
Langford 2011[72]	D	H	H.1	Asia	No	W, UI	W, C	Gender unequal	NA	NA	Gender unequal
Lee 1991[73]	D	H	H.0	Asia	No	W, UI	W, OSI	Gender unequal	NA	NA	Gender unequal
Lindquist 2014[74]	D	W	W.5	Latin America	No	W, UI	W	Gender unequal	Gender unequal	NA	NA

(Supplementary Table 1 Continued)

Study ID	SRMA source[a]	Int. Focus[b]	Int. Type[c]	Study region	Sex-disaggregated outcome data reported	Individuals targeted for intervention activities[d]	Individuals engaged for research activities[d]	GRAS overall	GRAS water int.	GRAS san. int.	GRAS hygiene int.
Liu 2019[75]	ARI	H	H.1	Asia	No	C, OSI, UI	C, OSI	Gender unaware	NA	NA	Gender unaware
Luby 2004[76]	D	W, H	W.5, H.1	Asia	No	UI	W, W or M, UI	Gender unaware	Gender unaware	NA	Gender unaware
Luby 2004* [77]	D	H	H.1	Asia	No	UI	W, C, UI	Gender unaware	NA	NA	Gender unaware
Luby 2005* [78]	ARI	H	H.1	Asia	No	W, M, UI	W, C	Gender unequal	NA	NA	Gender unequal
Luby 2006[79]	D	W, H	W.5, H.1	Asia	No	OSI, UI	W	Gender unaware	Gender unaware	NA	Gender unaware
Luby 2018[80]	D	W, S, H	W.5, S.2, H.1	Asia	No	W, C, UI	W, C	Gender unequal	Gender unaware	Gender unequal	Gender unaware
Lule 2005[81]	D	W	W.5	Africa	No	UI	UI	Gender unaware	Gender unaware	NA	NA
Ma 2 019[82]	D	H	H.0	Africa	No	W, UI	W	Gender unequal	NA	NA	Gender unequal
Maeusezahl 2009[83]	D	W	W.5	Latin America	No	W, UI	W, UI	Gender unequal	Gender unequal	NA	NA
Mahfouz 1995[84]	D	W	W.5	Middle East	No	OSI	C, UI	Gender unaware	Gender unaware	NA	NA
Majuru 2011[85]	D	W	W.4	Africa	No	None	W or M	NA	NA	NA	NA
Manaseki-Holland 2021[86]	D, ARI	H	H.0	Africa	No	W, UI	W	Gender unequal	NA	NA	Gender unequal
Mangkla-keree 2014[87]	ARI	H	H.0	Asia	Yes	C	C, OSI	Gender unaware	NA	NA	Gender unaware
McGuigan 2011[88]	D	W	W.5	Asia	No	W, OSI	W, OSI, UI	Gender unequal	Gender unequal	NA	NA

(Supplementary Table 1 Continued)

Study ID	SRMA source[a]	Int. Focus[b]	Int. Type[c]	Study region	Sex-disaggregated outcome data reported	Individuals targeted for intervention activities[d]	Individuals engaged for research activities[d]	GRAS overall	GRAS water int.	GRAS san. int.	GRAS hygiene int.
Mengistie 2013[89]	D	W	W.5	Africa	No	W, UI	W, UI	Gender unaware	Gender unaware	NA	NA
Moraes 2003[90]	D	S	S.1	Latin America	No	None	W	NA	NA	NA	NA
Morris 2018[91]	D	W	W.5	Africa	No	UI	W, C, UI	Gender unaware	Gender unaware	NA	NA
Morse 2020[92]	D, ARI	H	H.0	Africa	No	W, UI	W	Gender unequal	NA	NA	Gender unequal
Najnin 2019[93]	ARI	H	H.1	Asia	No	UI	UI	Gender unaware	NA	NA	Gender unaware
Nicholson 2014[94]	D, ARI	H	H.1	Asia	No	W, C	W, UI	Gender unequal	NA	NA	Gender unequal
Null 2018[95]	D	W, S, H	W.5, S.2, H.1	Africa	No	W, OSI, UI	W, C, UI	Gender unaware	Gender unaware	Gender unaware	Gender unaware
Opryszko 2010[96]	D	W, H	W.5, H.1	Asia	No	W	W, W or M, UI	Gender unequal	Gender unequal	NA	Gender unequal
Patel 2012[97]	D, ARI	H	H.1	Africa	No	C, OSI	W, C	Gender unaware	NA	NA	Gender unaware
Patil 2014[98]	D	S	S.2	Africa	No	C, UI	W, C, UI	Gender unaware	NA	Gender unaware	NA
Pickering 2013[99]	D, ARI	H	H.1	Africa	No	C, OSI	C, OSI	Gender unaware	NA	NA	Gender unaware
Pickering 2015[100]	D, ARI	S, H	S.2, H.0	Africa	No	UI	W, C, UI	Gender unaware	NA	Gender unaware	Gender unaware
Pickering 2019[101]	D	W	W.2	Asia	No	OSI, UI	W, UI	Gender unaware	Gender unaware	NA	NA
Pinfold 1996[102]	D	H	H.1	Asia	No	W, C, OSI, UI	W, UI	Gender unequal	NA	NA	Gender unequal
Pradhan 2002[103]	D	S	S.1	Latin America	No	None	UI	NA	NA	NA	NA

(Supplementary Table 1 Continued)

Study ID	SRMA source[a]	Int. Focus[b]	Int. Type[c]	Study region	Sex-disaggregated outcome data reported	Individuals targeted for intervention activities[d]	Individuals engaged for research activities[d]	GRAS overall	GRAS water int.	GRAS san. int.	GRAS hygiene int.
Quick 1999[104]	D	W	W.5	Latin America	No	W, UI	W, W or M, C, UI	Gender unequal	Gender unequal	NA	NA
Quick 2002[105]	D	W	W.5	Africa	No	UI	W, UI	Gender unaware	Gender unaware	NA	NA
Rai 2010[106]	D	W	W.5	Asia	No	W	UI	Gender unequal	Gender unequal	NA	NA
Ram 2015[107]	ARI	H	H.1	Asia	No	UI	W, UI	Gender unaware	NA	NA	Gender unaware
Reese 2019[108]	D	W, S	W.3, S.2	Asia	No	UI	W, C, UI	Gender unaware	Gender unaware	Gender unaware	NA
Reller 2003[109]	D	W	W.5	Latin America	No	W	W, UI	Gender unequal	Gender unequal	NA	NA
Roberts 2000[110]	D	H	H.0	Oceana	No	C, OSI	C, OSI	Gender unaware	NA	NA	Gender unaware
Rose 2006[111]	D	W	W.5	Asia	No	W	W	Gender unequal	Gender unequal	NA	NA
Ryder 1985[112]	D	W	W.3	Latin America	No	None	C, UI	NA	NA	NA	NA
Semenza 1998[113]	D	W	W.5	Europe	No	UI	UI	Gender unaware	Gender unaware	NA	NA
Shahid 1996[114]	D	H	H.1	Asia	No	W, M, UI	C, UI	Gender unaware	NA	NA	Gender unaware
Simmer-man 2011[115]	ARI	H	H.1	Asia	No	UI	C, UI	Gender unaware	NA	NA	Gender unaware
Sinharoy 2017[116]	D	H	H.0	Africa	No	UI	W, C, UI	Gender unaware	NA	NA	Gender unaware
Sircar 1987[117]	D	H	H.1	Asia	No	C, UI	W, UI	Gender unaware	NA	NA	Gender unaware
Sobsey 2003[118]	D	W	W.5	Asia	No	UI	UI	Gender unaware	Gender unaware	NA	NA

(Supplementary Table 1 Continued)

Study ID	SRMA source[a]	Int. Focus[b]	Int. Type[c]	Study region	Sex-disaggregated outcome data reported	Individuals targeted for intervention activities[d]	Individuals engaged for research activities[d]	GRAS overall	GRAS water int.	GRAS san. int.	GRAS hygiene int.
Solomon 2020[119]	D	W	W.5	Africa	No	W	W, UI	Gender unequal	Gender unequal	NA	NA
Stanton 1988[120]	D	H	H.0	Asia	No	OSI, UI	W, C, UI	Gender unaware	NA	NA	Gender unaware
Stauber 2009[121]	D	W	W.5	Latin America	No	UI	W, UI	Gender unaware	Gender unaware	NA	NA
Stauber 2012[122]	D	W	W.5	Africa	No	UI	UI	Gender unaware	Gender unaware	NA	NA
Stauber 2012[123]	D	W	W.5	Asia	No	UI	W, UI	Gender unaware	Gender unaware	NA	NA
Swarthout 2020[124]	ARI	H	H.1	Africa	Yes	W, UI	W, C	Gender unequal	NA	NA	Gender unequal
Talaat 2011[125]	D, ARI	H	H.1	Middle East	No	C, OSI	C, OSI	Gender unaware	NA	NA	Gender unaware
Tiwari 2009[126]	D	W	W.5	Africa	No	W	W, UI	Gender unequal	Gender unequal	NA	NA
Tonglet 1992[127]	D	W	W.4	Latin America	No	None	W, UI	NA	NA	NA	NA
Trinies 2016[128]	ARI	H	H.1	Africa	No	C, OSI, UI	C, OSI	Gender unaware	NA	NA	Gender unaware
Universi-dad Rafael Landivar 1995[129]	D	W	W.5	Latin America	No	W, UI	W, C, UI	Gender unequal	Gender unequal	NA	NA
Walker 1999[130]	D	S	S.2	Latin America	No	None	W, M, OSI, UI	NA	NA	NA	NA
Wang 1989[131]	D	W	W.3	Asia	No	None	None	NA	NA	NA	NA
Wilson 1991[132]	D	H	H.1	Asia	No	W	W	Gender unequal	NA	NA	Gender unequal
Zomer 2015[133]	D	H	H.1	Europe	No	OSI	OSI	Gender unaware	NA	NA	Gender unaware

(Supplementary Table 1 Continued)

Footnotes

a. SRMA Source: D- Diarrhoea SRMA; ARI- Acute Respiratory Infection SRMA.

b. Intervention Focus: H- Hygiene; S- Sanitation; W- Water.

c. Intervention Type:
H.0- Promotion of handwashing without soap provision; H.1- Promotion of handwashing with soap provision;
S.1- Basic sanitation services without sewer connection; S.2- Basic sanitation services with sewer connection;
W.1- Improved, on premises, continuous supply; W.2- Improved on premises, higher WQ; W.3- Improved, on premises; W.4- Improved, not on premises;
W.5- Point-of-use treamtment of water from unimproved water source or improved source not on premises.

d. Individuals in research and intervention activities: C- Children; M- Man, W- Woman; OSI- Other Specified Individual; UI- Unspecified Individual.

*These two studies by Luby et al. report on the same intervention in two separate papers. These papers report on different health outcomes and were in different systematic reviews.

Supplementary Table 2: Examples of gender-unequal and gender-unaware water, sanitation, and hygiene intervention activities from included studies

	Gender unequal	Gender unaware
**Water**	To reduce childhood diarrhea, a solar drinking water disinfection intervention was implemented. In intervention households, field workers visited biweekly for a year to emphasize the use of solar disinfection. Mothers and primary caregivers were specifically targeted to integrate the water treatment into their daily life. Other promotion activities occurred with the entire community and primary schools on a less frequent basis (i.e., monthly and three times, respectively).[83]	To prevent child diarrhea, a household biosand filter intervention was implemented. Intervention households received concrete biosand filters, education on how to use and maintain the filter, and a five-gallon narrow mouth bottle and base that allowed water to filter directly into the container for safe storage. Some intervention households had two follow-up visits post-installation.[2]
**Sanitation**	To reduce child stunting and anemia, independent and combined water, sanitation, and hygiene interventions were implemented. Women were enrolled in the study and scheduled to receive 15 behavior-change modules from village health workers over 12 months based on their 'treatment' group assignment (i.e., standard of care; infant and young child feeding; water, sanitation, and hygiene; infant and young child feeding plus water, sanitation, and hygiene). A sequential longitudinal intervention was also delivered, and reviewed modules with mothers monthly for six months. Household sanitation and hygiene infrastructure was also built and chlorine for water treatment was distributed.[58]	To prevent diarrhea, soil-transmitted helminth infection, and child malnutrition, India's Total Sanitation Campaign was delivered, combining social mobilization and a post-hoc latrine subsidy. The Government of India provided subsidies for the construction of latrines that met specified criteria in below-poverty-line households. Motivators in every village were trained to help mobilize the community as a whole.[31]
**Hygiene**	To prevent childhood infections and growth faltering, a community-based handwashing program that targeted mothers was implemented. In intervention communities, the program was launched at a community meeting that included an interactive educational session, discussion, and short play. Then, community motivators conducted home visits with mothers to encourage the establishment of new handwashing routines for six months. Daily visits were conducted for two weeks, and then decreased in frequency until the mothers were visited just once or twice a week. Group meetings were also held for mothers in each study area every two weeks to promote handwashing behaviors and distribute new bars of soap.[72]	To prevent childhood diarrhea, an intervention to promote handwashing with soap after defecation and before preparing food, eating, and feeding a child was implemented. Field workers from local communities conducted weekly neighborhood meetings about handwashing for approximately one year, using slideshows, videotapes, and pamphlets. Field workers encouraged all household members who were old enough to understand to wash their hands at key times and provided soap to families, as needed. [77]

Supplementary Table 3: Prisma Checklist for Abstracts

Section and Topic	Item #	Checklist item	Reported (Yes/No)
**TITLE**			
Title	1	Identify the report as a systematic review.	Y
**BACKGROUND**			
Objectives	2	Provide an explicit statement of the main objective(s) or question(s) the review addresses.	Y
**METHODS**			
Eligibility criteria	3	Specify the inclusion and exclusion criteria for the review.	Y
Information sources	4	Specify the information sources (e.g. databases, registers) used to identify studies and the date when each was last searched.	Y
Risk of bias	5	Specify the methods used to assess risk of bias in the studies included.	NA; bias assessed in source systematic reviews
Synthesis of results	6	Specify the methods used to present and synthesise results.	Y
**RESULTS**			
Included studies	7	Give the total number of included studies and participants and summarise relevant characteristics of studies.	Y
Synthesis of results	8	Present results for main outcomes, preferably indicating the number of included studies and participants for each. If meta-analysis was done, report the summary estimate and confidence/credible interval. If comparing groups, indicate the direction of the effect (i.e. which group is favoured).	Y
**DISCUSSION**			
Limitations of evidence	9	Provide a brief summary of the limitations of the evidence included in the review (e.g. study risk of bias, inconsistency and imprecision).	Y
Interpretation	10	Provide a general interpretation of the results and important implications.	Y
**OTHER**			
Funding	11	Specify the primary source of funding for the review.	Y
Registration	12	Provide the register name and registration number.	Y

Supplementary Table 4: Prisma Checklist for Reviews

Section and Topic	Item #	Checklist item	Location where item is reported
**TITLE**			
Title	1	Identify the report as a systematic review.	Y
**ABSTRACT**			
Abstract	2	See the PRISMA 2020 for Abstracts checklist.	See Supplementary Table
**INTRODUCTION**			
Rationale	3	Describe the rationale for the review in the context of existing knowledge.	Introduction body
Objectives	4	Provide an explicit statement of the objective(s) or question(s) the review addresses.	Introduction: *final paragraph*
**METHODS**			
Eligibility criteria	5	Specify the inclusion and exclusion criteria for the review and how studies were grouped for the syntheses.	Methods: *Inclusion criteria and eligibility*
Information sources	6	Specify all databases, registers, websites, organisations, reference lists and other sources searched or consulted to identify studies. Specify the date when each source was last searched or consulted.	Methods: *Inclusion criteria and eligibility*
Search strategy	7	Present the full search strategies for all databases, registers and websites, including any filters and limits used.	Methods: *Inclusion criteria and eligibility*
Selection process	8	Specify the methods used to decide whether a study met the inclusion criteria of the review, including how many reviewers screened each record and each report retrieved, whether they worked independently, and if applicable, details of automation tools used in the process.	Methods: *Inclusion criteria and eligibility*
Data collection process	9	Specify the methods used to collect data from reports, including how many reviewers collected data from each report, whether they worked independently, any processes for obtaining or confirming data from study investigators, and if applicable, details of automation tools used in the process.	Methods: *Data extraction*
Data items	10a	List and define all outcomes for which data were sought. Specify whether all results that were compatible with each outcome domain in each study were sought (e.g. for all measures, time points, analyses), and if not, the methods used to decide which results to collect.	Methods: *Data extraction*
	10b	List and define all other variables for which data were sought (e.g. participant and intervention characteristics, funding sources). Describe any assumptions made about any missing or unclear information.	Methods: *Data extraction*
Study risk of bias assessment	11	Specify the methods used to assess risk of bias in the included studies, including details of the tool(s) used, how many reviewers assessed each study and whether they worked independently, and if applicable, details of automation tools used in the process.	NA
Effect measures	12	Specify for each outcome the effect measure(s) (e.g. risk ratio, mean difference) used in the synthesis or presentation of results.	NA

Synthesis methods	13a	Describe the processes used to decide which studies were eligible for each synthesis (e.g. tabulating the study intervention characteristics and comparing against the planned groups for each synthesis (item #5)).	Methods: *Data extraction*
	13b	Describe any methods required to prepare the data for presentation or synthesis, such as handling of missing summary statistics, or data conversions.	NA
	13c	Describe any methods used to tabulate or visually display results of individual studies and syntheses.	Methods: *Analysis*
	13d	Describe any methods used to synthesize results and provide a rationale for the choice(s). If meta-analysis was performed, describe the model(s), method(s) to identify the presence and extent of statistical heterogeneity, and software package(s) used.	Methods: *Analysis*
	13e	Describe any methods used to explore possible causes of heterogeneity among study results (e.g. subgroup analysis, meta-regression).	NA
	13f	Describe any sensitivity analyses conducted to assess robustness of the synthesized results.	NA
Reporting bias assessment	14	Describe any methods used to assess risk of bias due to missing results in a synthesis (arising from reporting biases).	NA
Certainty assessment	15	Describe any methods used to assess certainty (or confidence) in the body of evidence for an outcome.	NA
**RESULTS**			
Study selection	16a	Describe the results of the search and selection process, from the number of records identified in the search to the number of studies included in the review, ideally using a flow diagram.	Results: *paragraph 1*; Supplementary Figure 1
	16b	Cite studies that might appear to meet the inclusion criteria, but which were excluded, and explain why they were excluded.	Results: *paragraph 1*; Supplementary Figure 1
Study characteristics	17	Cite each included study and present its characteristics.	Supplementary Table 3
Risk of bias in studies	18	Present assessments of risk of bias for each included study.	NA
Results of individual studies	19	For all outcomes, present, for each study: (a) summary statistics for each group (where appropriate) and (b) an effect estimate and its precision (e.g. confidence/credible interval), ideally using structured tables or plots.	Tables 1,2, & 3 Supplementary Table 4 & 5
Results of syntheses	20a	For each synthesis, briefly summarise the characteristics and risk of bias among contributing studies.	NA
	20b	Present results of all statistical syntheses conducted. If meta-analysis was done, present for each the summary estimate and its precision (e.g. confidence/credible interval) and measures of statistical heterogeneity. If comparing groups, describe the direction of the effect.	Tables 1,2, & 3 Supplementary Table 4 & 5
	20c	Present results of all investigations of possible causes of heterogeneity among study results.	NA
	20d	Present results of all sensitivity analyses conducted to assess the robustness of the synthesized results.	NA

Reporting biases	21	Present assessments of risk of bias due to missing results (arising from reporting biases) for each synthesis assessed.	NA
Certainty of evidence	22	Present assessments of certainty (or confidence) in the body of evidence for each outcome assessed.	NA
**DISCUSSION**			
Discussion	23a	Provide a general interpretation of the results in the context of other evidence.	Discussion, paragraph 1
	23b	Discuss any limitations of the evidence included in the review.	Discussion, last paragraph
	23c	Discuss any limitations of the review processes used.	Discussion, last paragraph
	23d	Discuss implications of the results for practice, policy, and future research.	Discussion, throughout
**OTHER INFORMATION**			
Registration and protocol	24a	Provide registration information for the review, including register name and registration number, or state that the review was not registered.	Methods: paragraph 1
	24b	Indicate where the review protocol can be accessed, or state that a protocol was not prepared.	Methods: paragraph 1
	24c	Describe and explain any amendments to information provided at registration or in the protocol.	NA
Support	25	Describe sources of financial or non-financial support for the review, and the role of the funders or sponsors in the review.	Methods: *Role of funder*
Competing interests	26	Declare any competing interests of review authors.	Will be noted in separate 'Declaration of interests' Section
Availability of data, code and other materials	27	Report which of the following are publicly available and where they can be found: template data collection forms; data extracted from included studies; data used for all analyses; analytic code; any other materials used in the review.	Will be included as supplements and/or posted on OSF

Supplementary Text 1: Reflexivity Statement

The training and experience of the authors have informed this re-review. Specifically, all authors have experience—be it training, research, practice, or a combination thereof—in both water, sanitation and hygiene (WASH) and public health. Indeed, it is this shared WASH-public health lens that informed the specific scope of the re-review and the methodological approach for carrying it out. We acknowledge that we are all at institutions based in high income countries, which may have shaped our lens. Several of the authors bring additional expertise that has informed this re-review, as outlined below.

Several of the co-authors have co-authored other WASH and/or health- related systematic reviews, informing the overall approach to this re-review (AB, BC, OC, MP, IR, JS, SS, JW).

Some of the authors co-authored one or both of the systematic reviews from which the papers re-reviewed herein were sampled. This involvement was critical in shaping our understanding of critical gaps in these systematic reviews and in informing the portion of the analysis that engaged the exposure scenarios. Specifically, BC, OC, and JW were co-authors on the systematic review that assessed the effectiveness of water, sanitation, and handwashing with soap interventions on diarrheal disease (JW was lead author),[1] and OC and JW were co-authors on the systematic review that assessed the effectiveness of handwashing with soap for preventing acute respiratory infections. [2]

Some of the authors also have been engaged in large scale randomized control trials that have sought to understand the impact of WASH interventions (BC, OC, SS) on health and health-related behavioral outcomes, like those included in the analysis sample for this re-review. In fact, OC and SS are authors on some of the studies examined in this re-review. Involvement in these and similar trials provided insight regarding data collection, and therefore informed and inspired the need to assess how individuals are engaged in both intervention delivery and data collection.

Several of the authors bring experience carrying out research at the intersection of gender, WASH, and health (AB, BC, MP, IR, SS), and some bring experience in gender that extends beyond WASH and health (IR, SS). The experience examining gender in the context of WASH and health informed the use of the WHO Gender Responsiveness Assessment Scale, and the study's framing in the introduction and the findings in the discussion.

1. Wolf J, Johnston RB, Ambelu A, et al. Burden of disease attributable to unsafe drinking water, sanitation, and hygiene in domestic settings: a global analysis for selected adverse health outcomes. *The Lancet 2023*.

2. Ross I, Bick S, Ayieko P, et al. Effectiveness of handwashing with soap for preventing acute respiratory infections in low-income and middle-income countries: a systematic review and meta-analysis. *The Lancet* 2023.

Supplementary References 1. Full Citations for All Included Studies

1. Ahmed NU, Zeitlin MF, Beiser AS, Super CM, Gershoff SN. A longitudinal study of the impact of behavioural change intervention on cleanliness, diarrhoeal morbidity and growth of children in rural Bangladesh. *Soc Sci Med* 1993; **37**(2): 159-71.
2. Aiken BA, Stauber CE, Ortiz GM, Sobsey MD. An assessment of continued use and health impact of the concrete biosand filter in Bonao, Dominican Republic. *The American Journal of Tropical Medicine and Hygiene* 2011; **85**(2): 309.
3. Alam N, Wojtyniak B, Henry FJ, Rahaman MM. Mothers' personal and domestic hygiene and diarrhoea incidence in young children in rural Bangladesh. *Int J Epidemiol* 1989; **18**(1): 242-7.
4. Arnold B, Arana B, Mäusezahl D, Hubbard A, Colford Jr JM. Evaluation of a pre-existing, 3-year household water treatment and handwashing intervention in rural Guatemala. *Int J Epidemiol* 2009; **38**(6): 1651-61.
5. Arnold BF, Khush RS, Ramaswamy P, et al. Causal inference methods to study nonrandomized, preexisting development interventions. *Proceedings of the National Academy of Sciences* 2010; **107**(52): 22605-10.
6. Ashraf S, Islam M, Unicomb L, et al. Effect of Improved Water Quality, Sanitation, Hygiene and Nutrition Interventions on Respiratory Illness in Young Children in Rural Bangladesh: A Multi-Arm Cluster-Randomized Controlled Trial. *Am J Trop Med Hyg* 2020; **102**(5): 1124-30.
7. Austin C. Investigation of in-house water chlorination and its effectiveness for rural areas of the Gambia [dissertation]. *New Orleans: Tulane University School of Public Health and Tropical Medicine* 1993.
8. Aziz KMA, Hoque BA, Hasan KZ, et al. Reduction in diarrhoeal diseases in children in rural Bangladesh by environmental and behavioural modifications. *Trans R Soc Trop Med Hyg* 1990; **84**(3): 433-8.
9. Azor-Martinez E, Garcia-Fernandez L, Strizzi JM, et al. Effectiveness of a hand hygiene program to reduce acute gastroenteritis at child care centers: A cluster randomized trial. *Am J Infect Control* 2020; **48**(11): 1315-21.
10. Ban HQ, Tao L, Jin S, et al. Effects of multiple cleaning and disinfection interventions on infectious diseases in children: a group randomized trial in China. *Biomedical and Environmental Sciences* 2015; **28**(11): 779-87.
11. Bartlett AV, Jarvis BA, Ross V, et al. Diarrheal illness among infants and toddlers in day care centers: effects of active surveillance and staff training without subsequent monitoring. *Am J Epidemiol* 1988; **127**(4): 808-17.
12. Begum MR, Al Banna MH, Akter S, et al. Effectiveness of wash education to prevent diarrhea among children under five in a community of Patuakhali, Bangladesh. *SN Comprehensive Clinical Medicine* 2020; **2**: 1158-62.
13. Bitew BD, Gete YK, Biks GA, Adafrie TT. The effect of SODIS water treatment intervention at the household level in reducing diarrheal incidence among children under 5 years of age: a cluster randomized controlled trial in Dabat district, northwest Ethiopia. *Trials* 2018; **19**: 1-15.
14. Black RE, Dykes AC, Anderson KE, et al. Handwashing to prevent diarrhea in day-care centers. *Am J Epidemiol* 1981; **113**(4): 445-51.
15. Boisson S, Stevenson M, Shapiro L, et al. Effect of household-based drinking water chlorination on diarrhoea among children under five in Orissa, India: a double-blind randomised placebo-controlled trial. *PLoS Med* 2013; **10**(8): e1001497.

16. Bowen A, Ma H, Ou J, et al. A cluster-randomized controlled trial evaluating the effect of a handwashing-promotion program in Chinese primary schools. *Am J Trop Med Hyg* 2007; **76**(6): 1166-73.
17. Briceño B, Coville A, Martinez S. Promoting handwashing and sanitation: evidence from a large-scale randomized trial in rural Tanzania. *World Bank Policy Research Working Paper* 2015; (7164).
18. Brown J, Sobsey M. Improving household drinking water quality use of ceramic water filters in Cambodia: UNICEF; 2007.
19. Brown J, Sobsey MD, Loomis D. Local drinking water filters reduce diarrheal disease in Cambodia: a randomized, controlled trial of the ceramic water purifier. *The American Journal of Tropical Medicine and Hygiene* 2008; **79**(3): 394-400.
20. Brown J, Hien VT, McMahan L, et al. Relative benefits of on-plot water supply over other 'improved'sources in rural Vietnam. *Trop Med Int Health* 2013; **18**(1): 65-74.
21. Butz AM, Larson E, Fosarelli P, Yolken R. Occurrence of infectious symptoms in children in day care homes. *Am J Infect Control* 1990; **18**(6): 347-53.
22. Carabin H, Gyorkos TW, Soto JC, Joseph L, Payment P, Collet J-P. Effectiveness of a training program in reducing infections in toddlers attending day care centers. *Epidemiology* 1999; **10**(3): 219-27.
23. Cha S, Kang D, Tuffuor B, et al. The effect of improved water supply on diarrhea prevalence of children under five in the Volta region of Ghana: a cluster-randomized controlled trial. *Int J Env Res Public Health* 2015; **12**(10): 12127-43.
24. Chard AN, Garn JV, Chang HH, Clasen T, Freeman MC. Impact of a school-based water, sanitation, and hygiene intervention on school absence, diarrhea, respiratory infection, and soil-transmitted helminths: results from the WASH HELPS cluster-randomized trial. *J Glob Health* 2019; **9**(2).
25. Chase C, Do Q-T. Handwashing behavior change at scale: evidence from a randomized evaluation in Vietnam. *World Bank Policy Research Working Paper* 2012; (6207).
26. Chiller TM, Mendoza CE, Lopez MB, et al. Reducing diarrhoea in Guatemalan children: randomized controlled trial of flocculant-disinfectant for drinking-water. *Bull WHO* 2006; **84**(1): 28-35.
27. Chongsuvivatwong V, Mo-Suwan L, Chompikul J, Vitsupakorn K, McNeil D. Effects of piped water supply on the incidence of diarrheal diseases in children in southern Thailand. *Southeast Asian J Trop Med Public Health* 1994; **25**(4): 628-32.
28. Clasen TF, Brown J, Collin S, Suntura O, Cairncross S. Reducing diarrhea through the use of household-based ceramic water filters: a randomized, controlled trial in rural Bolivia. *The American Journal of Tropical Medicine and Hygiene* 2004; **70**(6): 651-7.
29. Clasen T, Garcia Parra G, Boisson S, Collin S. Household-based ceramic water filters for the prevention of diarrhea: a randomized, controlled trial of a pilot program in Colombia. *The American Journal of Tropical Medicine and Hygiene* 2005; **73**(4): 790-5.
30. Clasen TF, Brown J, Collin SM. Preventing diarrhoea with household ceramic water filters: assessment of a pilot project in Bolivia. *Int J Environ Health Res* 2006; **16**(03): 231-9.
31. Clasen T, Boisson S, Routray P, et al. Effectiveness of a rural sanitation programme on diarrhoea, soil-transmitted helminth infection, and child malnutrition in Odisha, India: a cluster-randomised trial. *The Lancet Global Health* 2014; **2**(11): e645-e53.
32. Conroy RM, Elmore-Meegan M, Joyce T, McGuigan KG, Barnes J. Solar disinfection of drinking water and diarrhoea in Maasai children: a controlled field trial. *Lancet* 1996; **348**(9043): 1695-7.
33. Conroy RM, Meegan ME, Joyce T, McGuigan K, Barnes J. Solar disinfection of water reduces diarrhoeal disease: an update. *Arch Dis Child* 1999; **81**(4): 337-8.
34. Crump JA, Otieno PO, Slutsker L, et al. Household based treatment of drinking water with flocculant-disinfectant for preventing diarrhoea in areas with turbid source water in rural western Kenya: cluster randomised controlled trial. *The BMJ* 2005; **331**(7515): 478.
35. Devoto F, Duflo E, Dupas P, Parienté W, Pons V. Happiness on tap: Piped water adoption in urban Morocco. *American Economic Journal: Economic Policy* 2012; **4**(4): 68-99.

36. Dickinson KL, Patil SR, Pattanayak SK, Poulos C, Yang J-H. Nature's call: impacts of sanitation choices in Orissa, India. *Econ Dev Cult Change* 2015; **64**(1): 1-29.
37. du Preez M, Conroy RM, Wright JA, Moyo S, Potgieter N, Gundry SW. Use of ceramic water filtration in the prevention of diarrheal disease: a randomized controlled trial in rural South Africa and Zimbabwe. *Am J Trop Med Hyg* 2008; **79**(5): 696.
38. du Preez M, McGuigan KG, Conroy RM. Solar disinfection of drinking water in the prevention of dysentery in South African children aged under 5 years: the role of participant motivation. *Environ Sci Technol* 2010; **44**(22): 8744-9.
39. du Preez M, Conroy RM, Ligondo S, et al. Randomized intervention study of solar disinfection of drinking water in the prevention of dysentery in Kenyan children aged under 5 years. *Environ Sci Technol* 2011; **45**(21): 9315-23.
40. Ercumen A, Arnold F, Kumpel E, et al. Upgrading a piped water supply from intermittent to continuous delivery and association with waterborne illness: a matched cohort study in urban India. *PLoS Med* 2015; **12**(10): e1001892.
41. Ercumen A, Naser AM, Unicomb L, Arnold BF, Colford Jr JM, Luby SP. Effects of source-versus household contamination of tubewell water on child diarrhea in rural Bangladesh: a randomized controlled trial. *PLoS One* 2015; **10**(3): e0121907.
42. Fabiszewski de Aceituno AM, Stauber CE, Walters AR, Sanchez REM, Sobsey MD. A randomized controlled trial of the plastic-housing BioSand filter and its impact on diarrheal disease in Copan, Honduras. *The American Journal of Tropical Medicine and Hygiene* 2012; **86**(6): 913.
43. Fagerli K, Gieraltowski L, Nygren B, et al. Use, acceptability, performance, and health impact of hollow fiber ultrafilters for water treatment in rural Kenyan households, 2009–2011. *The American Journal of Tropical Medicine and Hygiene* 2020; **103**(1): 465.
44. Galiani S, Gonzalez-Rozada M, Schargrodsky E. Water expansions in shantytowns: Health and savings. *Economica* 2009; **76**(304): 607-22.
45. Galiani S, Gertler P, Ajzenman N, Orsola-Vidal A. Promoting handwashing behavior: The effects of large-scale community and school-level interventions. *Health Economics* 2015; **25**(12): 1545-59.
46. Garrett V, Ogutu P, Mabonga P, et al. Diarrhoea prevention in a high-risk rural Kenyan population through point-of-use chlorination, safe water storage, sanitation, and rainwater harvesting. *Epidemiology & Infection* 2008; **136**(11): 1463-71.
47. Gasana J, Morin J, Ndikuyeze A, Kamoso P. Impact of water supply and sanitation on diarrheal morbidity among young children in the socioeconomic and cultural context of Rwanda (Africa). *Environ Res* 2002; **90**(2): 76-88.
48. Graf J, Zebaze Togouet S, Kemka N, Niyitegeka D, Meierhofer R, Gangoue Pieboji J. Health gains from solar water disinfection (SODIS): evaluation of a water quality intervention in Yaounde, Cameroon. *J Water Health* 2010; **8**(4): 779-96.
49. Gruber JS, Reygadas F, Arnold BF, Ray I, Nelson K, Colford Jr JM. A stepped wedge, cluster-randomized trial of a household UV-disinfection and safe storage drinking water intervention in rural Baja California Sur, Mexico. *The American Journal of Tropical Medicine and Hygiene* 2013; **89**(2): 238.
50. Haggerty PA, Muladi K, Kirkwood BR, Ashworth A, Manunebo M. Community-based hygiene education to reduce diarrhoeal disease in rural Zaire: impact of the intervention on diarrhoeal morbidity. *Int J Epidemiol* 1994; **23**(5): 1050-9.
51. Hammer J, Spears D. Village sanitation and child health: Effects and external validity in a randomized field experiment in rural India. *J Health Econ* 2016; **48**: 135-48.
52. Han AM, Hlaing T. Prevention of diarrhoea and dysentery by hand washing. *Trans R Soc Trop Med Hyg* 1989; **83**(1): 128-31.

53. Harshfield E, Lantagne D, Turbes A, Null C. Evaluating the sustained health impact of household chlorination of drinking water in rural Haiti. *The American Journal of Tropical Medicine and Hygiene* 2012; **87**(5): 786.
54. Hartinger SM, Lanata CF, Hattendorf J, et al. Improving household air, drinking water and hygiene in rural Peru: a community-randomized–controlled trial of an integrated environmental home-based intervention package to improve child health. *Int J Epidemiol* 2016; **45**(6): 2089-99.
55. Hashi A, Kumie A, Gasana J. Hand washing with soap and WASH educational intervention reduces under-five childhood diarrhoea incidence in Jigjiga District, Eastern Ethiopia: a community-based cluster randomized controlled trial. *Preventive Medicine Reports* 2017; **6**: 361-8.
56. Hill CL, McCain K, Nyathi ME, et al. Impact of low-cost point-of-use water treatment technologies on enteric infections and growth among children in Limpopo, South Africa. *The American Journal of Tropical Medicine and Hygiene* 2020; **103**(4): 1405.
57. Huda TMN, Unicomb L, Johnston RB, Halder MK, Sharker MAY, Luby SP. Interim evaluation of a large scale sanitation, hygiene and water improvement programme on childhood diarrhea and respiratory disease in rural Bangladesh. *Soc Sci Med* 2012; **75**(4): 604-11.
58. Humphrey JH, Mbuya MN, Ntozini R, et al. Independent and combined effects of improved water, sanitation, and hygiene, and improved complementary feeding, on child stunting and anaemia in rural Zimbabwe: a cluster-randomised trial. *The Lancet Global Health* 2019; **7**(1): e132-e47.
59. Hunter PR, Ramírez Toro GI, Minnigh HA. Impact on diarrhoeal illness of a community educational intervention to improve drinking water quality in rural communities in Puerto Rico. *BMC Public Health* 2010; **10**: 1-11.
60. Hussam R, Rabbani A, Reggiani G, Rigol N. Rational habit formation: experimental evidence from handwashing in India. *American Economic Journal: Applied Economics* 2022; **14**(1): 1-41.
61. Iijima Y, Karama M, Oundo JO, Honda T. Prevention of bacterial diarrhea by pasteurization of drinking water in Kenya. *Microbiol Immunol* 2001; **45**(6): 413-6.
62. Jain S, Sahanoon OK, Blanton E, et al. Sodium dichloroisocyanurate tablets for routine treatment of household drinking water in periurban Ghana: a randomized controlled trial. *The american journal of tropical medicine and hygiene* 2010; **82**(1): 16.
63. Jensen PK, Ensink JHJ, Jayasinghe G, van der Hoek W, Cairncross S, Dalsgaard A. Effect of chlorination of drinking-water on water quality and childhood diarrhoea in a village in Pakistan. *Journal of Health, Population and Nutrition* 2003; **21**(1): 26-31.
64. Kirby MA, Nagel CL, Rosa G, et al. Use, microbiological effectiveness and health impact of a household water filter intervention in rural Rwanda—A matched cohort study. *Int J Hyg Environ Health* 2017; **220**(6): 1020-9.
65. Kirby MA, Nagel CL, Rosa G, et al. Effects of a large-scale distribution of water filters and natural draft rocket-style cookstoves on diarrhea and acute respiratory infection: a cluster-randomized controlled trial in Western Province, Rwanda. *PLoS Med* 2019; **16**(6): e1002812.
66. Kirchhoff LV, McClelland KE, Pinho MDC, Araujo JG, De Sousa MA, Guerrant RL. Feasibility and efficacy of in-home water chlorination in rural North-eastern Brazil. *Epidemiology & Infection* 1985; **94**(2): 173-80.
67. Klasen S, Lechtenfeld T, Meier K, Rieckmann J. Benefits trickling away: the health impact of extending access to piped water and sanitation in urban Yemen. *Journal of Development Effectiveness* 2012; **4**(4): 537-65.
68. Kolahi A-A, Rastegarpour A, Sohrabi M-R. The impact of an urban sewerage system on childhood diarrhoea in Tehran, Iran: a concurrent control field trial. *Trans R Soc Trop Med Hyg* 2009; **103**(5): 500-5.
69. Kotch JB, Faircloth AH, Weigle KA, et al. Evaluation of an hygienic intervention in child day-care centers. *Pediatrics* 1994; **94**(6): 991-4.

70. Kotch JB, Isbell P, Weber DJ, et al. Hand-washing and diapering equipment reduces disease among children in out-of-home child care centers. *Pediatrics* 2007; **120**(1): e29-e36.
71. Kremer M, Leino J, Miguel E, Zwane AP. Spring cleaning: Rural water impacts, valuation, and property rights institutions. *The Quarterly Journal of Economics* 2011; **126**(1): 145-205.
72. Langford R, Lunn P, Brick CP. Hand-washing, subclinical infections, and growth: A longitudinal evaluation of an intervention in Nepali slums. *American Journal of Human Biology* 2011; **23**(5): 621-9.
73. Lee W, Stoeckel J, Jintaganont P, Romanarak T, Kullavanijaya S. The impact of a community based health education program on the incidence of diarrheal disease in southern Thailand. *Southeast Asian J Trop Med Public Health* 1991; **22**(4): 548-56.
74. Lindquist ED, George CM, Perin J, et al. A cluster randomized controlled trial to reduce childhood diarrhea using hollow fiber water filter and/or hygiene–sanitation educational interventions. *The American Journal of Tropical Medicine and Hygiene* 2014; **91**(1): 190.
75. Liu X, Hou W, Zhao Z, et al. A hand hygiene intervention to decrease hand, foot and mouth disease and absence due to sickness among kindergarteners in China: A cluster-randomized controlled trial. *J Infect* 2019; **78**(1): 19-26.
76. Luby SP, Agboatwalla M, Hoekstra RM, Rahbar MH, Billhimer W, Keswick BH. Delayed effectiveness of home-based interventions in reducing childhood diarrhea, Karachi, Pakistan. *The American Journal of Tropical Medicine and Hygiene* 2004; **71**(4): 420-7.
77. Luby SP, Agboatwalla M, Painter J, Altaf A, Billhimer WL, Hoekstra RM. Effect of intensive handwashing promotion on childhood diarrhea in high-risk communities in Pakistan: a randomized controlled trial. *Jama* 2004; **291**(21): 2547-54.
78. Luby SP, Agboatwalla M, Feikin DR, et al. Effect of handwashing on child health: a randomised controlled trial. *Lancet* 2005; **366**(9481): 225-33.
79. Luby SP, Agboatwalla M, Painter J, et al. Combining drinking water treatment and hand washing for diarrhoea prevention, a cluster randomised controlled trial. *Trop Med Int Health* 2006; **11**(4): 479-89.
80. Luby SP, Rahman M, Arnold BF, et al. Effects of water quality, sanitation, handwashing, and nutritional interventions on diarrhoea and child growth in rural Bangladesh: a cluster randomised controlled trial. *Lancet Glob Health* 2018; **6**(3): e302-e15.
81. Lule JR, Mermin J, Ekwaru JP, et al. Effect of home based water chlorination and safe storage on diarrhea among persons with human immonodeficiency virus in Uganda. *Am J Trop Med Hyg* 2005; **73**(5): 926-33.
82. Ma Y, Sudfeld CR, Kim H, et al. Evaluating the impact of community health volunteer home visits on child diarrhea and fever in the Volta Region, Ghana: A cluster-randomized controlled trial. *PLoS Med* 2019; **16**(6): e1002830.
83. Mäusezahl D, Christen A, Pacheco GD, et al. Solar drinking water disinfection (SODIS) to reduce childhood diarrhoea in rural Bolivia: a cluster-randomized, controlled trial. *PLoS medicine* 2009; **6**(8): e1000125.
84. Mahfouz AAR, Abdel-Moneim M, Al-Erian RAG. Impact of chlorination of water in domestic storage tanks on childhood diarrhoea: a community trial in the rural areas. *Journal of Tropical Medicine and Hygiene* 1995; **98**(2): 126-30.
85. Majuru B, Mokoena MM, Jagals P, Hunter PR. Health impact of small-community water supply reliability. *Int J Hyg Environ Health* 2011; **214**(2): 162-6.
86. Manaseki-Holland S, Manjang B, Hemming K, et al. Effects on childhood infections of promoting safe and hygienic complementary-food handling practices through a community-based programme: A cluster randomised controlled trial in a rural area of The Gambia. *PLoS Med* 2021; **18**(1): e1003260.
87. Mangklakeree N, Pinitsoontorn S, Srisaenpang S. Effectiveness of influenza control using non-pharmaceutical interventions at primary schools in Nakhon Phanom province, northeast Thailand. *Asian Biomedicine* 2014; **8**(3): 405-10.

88. McGuigan KG, Samaiyar P, du Preez M, Conroy RM. High compliance randomized controlled field trial of solar disinfection of drinking water and its impact on childhood diarrhea in rural Cambodia. *Environ Sci Technol* 2011; **45**(18): 7862-7.
89. Mengistie B, Berhane Y, Worku A. Household water chlorination reduces incidence of diarrhea among under-five children in rural Ethiopia: a cluster randomized controlled trial. *PLoS One* 2013; **8**(10): e77887.
90. Moraes LRS, Cancio JA, Cairncross S, Huttly S. Impact of drainage and sewerage on diarrhoea in poor urban areas in Salvador, Brazil. *Trans R Soc Trop Med Hyg* 2003; **97**(2): 153-8.
91. Morris JF, Murphy J, Fagerli K, et al. A randomized controlled trial to assess the impact of ceramic water filters on prevention of diarrhea and cryptosporidiosis in infants and young children—Western Kenya, 2013. *The American Journal of Tropical Medicine and Hygiene* 2018; **98**(5): 1260.
92. Morse T, Tilley E, Chidziwisano K, Malolo R, Musaya J. Health outcomes of an integrated behaviour-centred water, sanitation, hygiene and food safety intervention–a randomised before and after trial. *Int J Env Res Public Health* 2020; **17**(8): 2648.
93. Najnin N, Leder K, Forbes A, et al. Impact of a large-scale handwashing intervention on reported respiratory illness: findings from a cluster-randomized controlled trial. *The American Journal of Tropical Medicine and Hygiene* 2019; **100**(3): 742.
94. Nicholson JA, Naeeni M, Hoptroff M, et al. An investigation of the effects of a hand washing intervention on health outcomes and school absence using a randomised trial in Indian urban communities. *Trop Med Int Health* 2014; **19**(3): 284-92.
95. Null C, Stewart CP, Pickering AJ, et al. Effects of water quality, sanitation, handwashing, and nutritional interventions on diarrhoea and child growth in rural Kenya: a cluster-randomised controlled trial. *Lancet Glob Health* 2018; **6**(3): e316-e29.
96. Opryszko MC, Majeed SW, Hansen PM, et al. Water and hygiene interventions to reduce diarrhoea in rural Afghanistan: a randomized controlled study. *J Water Health* 2010; **8**(4): 687-702.
97. Patel MK, Harris JR, Juliao P, et al. Impact of a hygiene curriculum and the installation of simple handwashing and drinking water stations in rural Kenyan primary schools on student health and hygiene practices. *The American Journal of Tropical Medicine and Hygiene* 2012; **87**(4): 594.
98. Patil SR, Arnold BF, Salvatore AL, et al. The effect of India's total sanitation campaign on defecation behaviors and child health in rural Madhya Pradesh: a cluster randomized controlled trial. *PLoS Med* 2014; **11**(8): e1001709.
99. Pickering AJ, Davis J, Blum AG, et al. Access to waterless hand sanitizer improves student hand hygiene behavior in primary schools in Nairobi, Kenya. *The American Journal of Tropical Medicine and Hygiene* 2013; **89**(3): 411.
100. Pickering AJ, Djebbari H, Lopez C, Coulibaly M, Alzua ML. Effect of a community-led sanitation intervention on child diarrhoea and child growth in rural Mali: a cluster-randomised controlled trial. *Lancet Glob Health* 2015; **3**(11): e701-e11.
101. Pickering AJ, Crider Y, Sultana S, et al. Effect of in-line drinking water chlorination at the point of collection on child diarrhoea in urban Bangladesh: a double-blind, cluster-randomised controlled trial. *Lancet Glob Health* 2019; **7**(9): e1247-e56.
102. Pinfold JV, Horan NJ. Measuring the effect of a hygiene behaviour intervention by indicators of behaviour and diarrhoeal disease. *Trans R Soc Trop Med Hyg* 1996; **90**(4): 366-71.
103. Pradhan M, Rawlings LB. The impact and targeting of social infrastructure investments: Lessons from the Nicaraguan Social Fund. *World Bank Economic Review* 2002; (16): 275-95.
104. Quick RE, Venczel LV, Mintz ED, et al. Diarrhoea prevention in Bolivia through point-of-use water treatment and safe storage: a promising new strategy. *Epidemiology & Infection* 1999; **122**(1): 83-90.

105. Quick RE, Kimura A, Thevos A, et al. Diarrhea prevention through household-level water disinfection and safe storage in Zambia. *The American Journal of Tropical Medicine and Hygiene* 2002; **66**(5): 584-9.
106. Rai BB, Pal R, Kar S, Tsering DC. Solar disinfection improves drinking water quality to prevent diarrhea in under-five children in Sikkim, India. *Journal of Global Infectious Diseases* 2010; **2**(3): 221-5.
107. Ram PK, DiVita MA, Khatun-e-Jannat K, et al. Impact of intensive handwashing promotion on secondary household influenza-like illness in rural Bangladesh: findings from a randomized controlled trial. *PLoS One* 2015; **10**(6): e0125200.
108. Reese H, Routray P, Torondel B, et al. Assessing longer-term effectiveness of a combined household-level piped water and sanitation intervention on child diarrhoea, acute respiratory infection, soil-transmitted helminth infection and nutritional status: a matched cohort study in rural Odisha, India. *Int J Epidemiol* 2019; **48**(6): 1757-67.
109. Reller ME, Mendoza CE, Lopez MB, et al. A randomized controlled trial of household-based flocculant-disinfectant drinking water treatment for diarrhea prevention in rural Guatemala. *Am J Trop Med Hyg* 2003; **69**(4): 411-9.
110. Roberts L, Jorm L, Patel M, Smith W, Douglas RM, McGilchrist C. Effect of infection control measures on the frequency of diarrheal episodes in child care: a randomized, controlled trial. *Pediatrics* 2000; **105**(4): 743-6.
111. Rose A, Roy S, Abraham V, et al. Solar disinfection of water for diarrhoeal prevention in southern India. *Arch Dis Child* 2006; **91**(2): 139-41.
112. Ryder RW, Reeves WC, Singh N, et al. The childhood health effects of an improved water supply system on a remote Panamanian island. *The American Journal of Tropical Medicine and Hygiene* 1985; **34**(5): 921-4.
113. Semenza JC, Roberts L, Henderson A, Bogan J, Rubin C. Water distribution system and diarrheal disease transmission: a case study in Uzbekistan. *The American journal of tropical medicine and hygiene* 1998; **59**(6): 941-6.
114. Shahid NS, Greenough III WB, Samadi AR, Huq MI, Rahman N. Hand washing with soap reduces diarrhoea and spread of bacterial pathogens in a Bangladesh village. *Journal of Diarrhoeal Diseases Research* 1996; **14**(2): 85-9.
115. Simmerman JM, Suntarattiwong P, Levy J, et al. Findings from a household randomized controlled trial of hand washing and face masks to reduce influenza transmission in Bangkok, Thailand. *Influenza and Other Respiratory Viruses* 2011; **5**(4): 256-67.
116. Sinharoy SS, Schmidt W-P, Wendt R, et al. Effect of community health clubs on child diarrhoea in western Rwanda: cluster-randomised controlled trial. *Lancet Glob Health* 2017; **5**(7): e699-e709.
117. Sircar BK, Sengupta PG, Mondal SK, et al. Effect of handwashing on the incidence of diarrhoea in a Calcutta slum. *Journal of Diarrhoeal Diseases Research* 1987; **5**(2): 112-4.
118. Sobsey MD, Handzel T, Venczel L. Chlorination and safe storage of household drinking water in developing countries to reduce waterborne disease. *Water Science and Technology* 2003; **47**(3): 221-8.
119. Solomon ET, Robele S, Kloos H, Mengistie B. Effect of household water treatment with chlorine on diarrhea among children under the age of five years in rural areas of Dire Dawa, eastern Ethiopia: a cluster randomized controlled trial. *Infectious Diseases of Poverty* 2020; **9**: 1-13.
120. Stanton BF, Clemens JD, Khair T. Educational intervention for altering water-sanitation behavior to reduce childhood diarrhea in urban Bangladesh: impact on nutritional status. *The American Journal of Clinical Nutrition* 1988; **48**(5): 1166-72.
121. Stauber CE, Ortiz GM, Loomis DP, Sobsey M. A randomized controlled trial of the concrete biosand filter and its impact on diarrheal disease in Bonao, Dominican Republic. *Am J Trop Med Hyg* 2009; **80**(2): 286-93.

122. Stauber CE, Kominek B, Liang KR, Osman MK, Sobsey MD. Evaluation of the impact of the plastic BioSand filter on health and drinking water quality in rural Tamale, Ghana. *Int J Env Res Public Health* 2012; **9**(11): 3806-23.
123. Stauber CE, Printy ER, McCarty FA, Liang KR, Sobsey MD. Cluster randomized controlled trial of the plastic BioSand water filter in Cambodia. *Environ Sci Technol* 2012; **46**(2): 722-8.
124. Swarthout J, Ram PK, Arnold CD, et al. Effects of individual and combined water, sanitation, handwashing, and nutritional interventions on child respiratory infections in rural Kenya: a cluster-randomized controlled trial. *The American Journal of Tropical Medicine and Hygiene* 2020; **102**(6): 1286.
125. Talaat M, Afifi S, Dueger E, et al. Effects of hand hygiene campaigns on incidence of laboratory-confirmed influenza and absenteeism in schoolchildren, Cairo, Egypt. *Emerging Infect Dis* 2011; **17**(4): 619.
126. Tiwari SSK, Schmidt WP, Darby J, Kariuki ZG, Jenkins MW. Intermittent slow sand filtration for preventing diarrhoea among children in Kenyan households using unimproved water sources: randomized controlled trial. *Trop Med Int Health* 2009; **14**(11): 1374-82.
127. Tonglet R, Isu K, Mpese M, Dramaix M, Hennart P. Can improvements in water supply reduce childhood diarrhoea? *Health Policy Plann* 1992; **7**(3): 260-8.
128. Trinies V, Garn JV, Chang HH, Freeman MC. The impact of a school-based water, sanitation, and hygiene program on absenteeism, diarrhea, and respiratory infection: a matched–control trial in Mali. *The American Journal of Tropical Medicine and Hygiene* 2016; **94**(6): 1418.
129. Universidad Rafael Landívar. Contra la morbilidad infantil: Filtros artesanales y educación: Universidad Rafael Landívar, Instituto de Investigaciones Económicas y Sociales; 1995.
130. Walker I, del Cid R, Ordoñez F, Rodríguez F. Ex-post evaluation of the Honduran social investment fund (FHIS 2). *ESA Consultores, Tegucigalpa, Honduras* 1999.
131. Wang ZS, Shepard DS, Zhu YC, et al. Reduction of enteric infectious disease in rural China by providing deep-well tap water. *Bull WHO* 1989; **67**(2): 171.
132. Wilson JM, Chandler GN. Hand-washing reduces diarrhoea episodes: a study in Lombok, Indonesia. *Trans R Soc Trop Med Hyg* 1991; **85**(6): 819-21.
133. Zomer TP, Erasmus V, Looman CW, et al. A hand hygiene intervention to reduce infections in child daycare: a randomized controlled trial. *Epidemiology & Infection* 2015; **143**(12): 2494-502.
